# Palladium(II)-Catalyzed Regioselective *Ortho* Arylation of sp^2^ C—H Bonds of *N*-Aryl-2-amino Pyridine Derivatives

**DOI:** 10.1002/cctc.201200155

**Published:** 2012-06-13

**Authors:** Moumita Koley, Navid Dastbaravardeh, Michael Schnürch, Marko D Mihovilovic

**Affiliations:** [a] Institute of Applied Synthetic Chemistry, Vienna University of TechnologyGetreidemarkt 9/163-OC, 1060 Vienna (Austria)

**Keywords:** amines, C—H activation, heterocycles, homogeneous catalysis, palladium

## Abstract

**Abstract:**

The direct arylation of *N*-(2-pyridyl) substituted anilines is described. Arylation takes place in *ortho* position to the amine functionality and is directed by the pyridine N-substituent. Remarkably, N-arylation was never observed as a competing process even though conditions also suitable for Buchwald–Hartwig reactions were applied. The scope of the reaction was investigated in terms of aryl donors as well as the electronic nature of the substrate. Good yields were obtained for most examples through an operationally simple procedure, which did not require inert conditions or even glove box techniques. Pd(OAc)_2_ was applied as a cheap catalyst and boronic acids as readily available aryl donors. To obtain full conversion, 1,4-benzoquinone and a silver salt (e.g., Ag_2_O) were required as additives and reacted at relatively mild temperatures (e.g., 80 °C). Additionally, the pyridine-directing group was cleaved after the reaction to give *ortho*-arylated aniline derivatives.

## Introduction

The formation of C—C bonds is a process of utmost importance in organic synthesis. Catalytic methods can realize such reactions. Prominent reactions in this regard are metal-catalyzed cross-coupling reactions of organo (pseudo) halides and various metal organyls.^1^ The great success of these reactions led to a Nobel Prize for Akira Suzuki, Richard Heck, and Ei-ichi Negishi in 2010. In recent years, a new trend has emerged in organic synthesis that attempts to further facilitate and simplify the transition metal catalyzed bond-forming process in synthetic strategy. Metal-catalyzed direct functionalization of C—H bonds (generally called C—H activation) strives to avoid at least one of the prefunctionalized building blocks typically used in cross-coupling reactions.^2^ This represents a significant advantage, as C—H bonds are ubiquitous in organic molecules and synthetic steps to obtain a required (pseudo) halide or organo-metal species can be avoided. Synthetic sequences become shorter and more time, resource, energy, and atom efficient, which falls perfectly in line with the principles of green chemistry.^3^ Such C—H activation reactions have been reported mainly for the functionalization of sp^3^ and sp^2^ C—H bonds (the Sonogashira reaction can be considered as a C—H activation reaction of an sp C—H bond). Methods to create C—C,^4^ C—O,^5^ C—N,^6^ C—S,^7^ and C—X^8^ bonds have been reported in recent years regarding the direct functionalization of sp^2^ hybridized C—H bonds. Since C—H bonds are omnipresent in organic molecules, this opens great opportunities for C—H activation chemistry, but also raises problems regarding the selectivity between this manifold of C—H bonds. Methods have been found to target one C—H bond of many to obtain a specific product from a direct functionalization reaction. The presence of a suitable directing group in the molecule is one way to achieve this: the metal catalyst is directed to a specific position, in which it activates one C—H bond. Regioselective functionalizations of C—H bonds have been reported on arenes by using directing groups, such as pyridine,^[9]^ pyridine *N*-oxide,4e oxazoline,^10^ isoxazole,^11^ carboxylic acids,^12^ anilides,^13^ aldehydes and ketones (or their imine derivatives),^14^ and amides,^15^ to name the most prominent ones. Although this provides a large arsenal of possible directing groups, most of these groups cannot be readily cleaved, which presents a major limitation. Palladium, ruthenium, and rhodium have been most frequently applied as catalysts. In an ongoing project to use pyridine as a directing group for the arylation of C—H bonds, we have demonstrated the feasibility of sp^3^ arylation under Ru-catalysis.^16^ Here, we report on the direct *ortho*-arylation of aniline derivatives directed by a 2-pyridyl N-substituent, which can also be cleaved after direct arylation.

## Results and Discussion

**Scheme 1:**
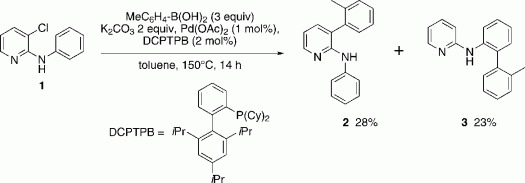
Initial finding of a pyridine directed direct arylation.

In our investigations towards regioselective and orthogonal cross-coupling methodologies on pyridine systems,^17^ an interesting and unexpected byproduct was observed, which clearly originated from a direct arylation after C—H activation (Scheme 1). Although the expected product of a Suzuki–Miyaura cross-coupling reaction in position 3 of pyridine was isolated as a major compound, *ortho*-arylation of the *N*-phenyl ring was also observed with a concomitant dechlorination. Dehalogenations in the presence of palladium are not uncommon, indeed they are exploited synthetically,^18^ however, the observed arylation represented an unusual and interesting result that indicated a directing effect by pyridine.

**Scheme 2:**
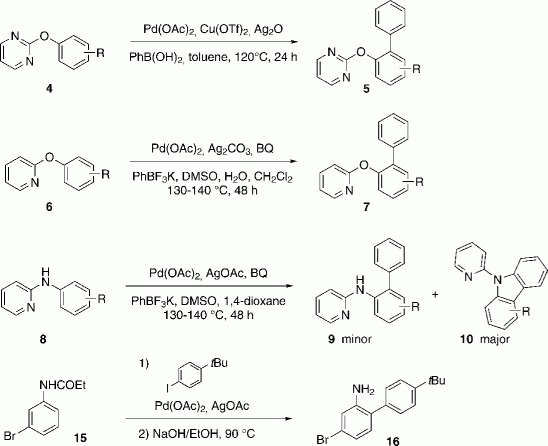
Literature procedures.

Remarkably, this transformation tolerated the presence of a free amino-group, as N-arylation would have also been possible under the applied reaction conditions. After scanning the literature for similar reactions, we found that *ortho*-arylation of 2-phenoxypyridines^19^ and 2-phenoxypyrimidines^20^ had been reported recently. In the case of 2-phenoxypyridines, potassium trifluoroborates were used as aryl donors, together with a complex mixture of solvents and additives (Scheme 2, **6** to **7**). Owing to the high reaction temperature of 130–140 °C, the reactions had to be performed in an autoclave and lasted typically for 48 h. In the reaction with 2-phenoxypyrimidines, the more convenient boronic acids could be used, as well as toluene as the sole solvent at 120 °C, which did not require the use of an autoclave (Scheme 2, **4**→**5**). The catalyst Pd(OAc)_2_ and the additives Ag_2_O and Cu(OTf)_2_ were used. Very recently, Wu and co-workers also reported a method with *N*-aryl-pyridine-2-amines as substrate (Scheme 2, **8**→**9**+**10**).^21^ Under their optimized conditions, arylation of the N-aryl substituent took place in the *ortho* position and the resultant intermediate reacted further to give the corresponding *N*-2-pyridyl-carbazoles **10** to form the major product. This showed the CH activation reaction under Pd catalysis in the presence of a free amino functionality to be challenging because C—N bond formation could also take place under these conditions. Daugulis and co-workers reported direct arylation of **15** in *ortho*-position to the carbamate group. After deprotection, *ortho*-arylated anilines **16** were obtained.13d Very recently, an Ru^II^-catalyzed protocol was also reported.^22^

Based on these reported procedures, we started an optimization effort to further exploit the secondary process observed in our initial experiments. It was quickly found that the condition successful in the arylation of 2-phenoxypyridines was not suitable for arylation of *N*-phenylpyridine-2-amines. The use of Ph—BF_3_K as aryl donor did not lead to the complete conversion of the starting material and biphenyl formation was the most prominent process (Table 1, entries 1 and 2). This side reaction was also the reason for the use of 2.5 equiv. of aryl donor in the literature.^19^ To disfavor homocoupling, the presence of oxygen was excluded by performing the reaction under an argon atmosphere, however no improvement was detected in this or other experiments that compared reactions in air to those in an inert atmosphere. On using a solvent that did not require the use of an autoclave, the conversion could only be increased to 61 % if toluene was used (entry 2). Use of 1,2-dichlorobenzene as solvent (DCB, *T*=140 and 180 °C) led to complete failure of the reaction; use of 1,2-dichloroethane (DCE) also proved inefficient (data not shown).

**Table 1:** Optimization of the pyridine directed direct arylation of 11 a.
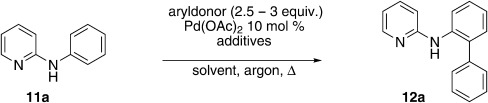

Entry	Solvent	*T* [°C]	Additives [equiv.]	Conversion^[a,b]^
1^[c]^	DCM	135	BQ (1.0) DMSO (4.0)	Ag_2_CO_3_ (2.0) H_2_O (8.0)	50
2^[c]^	toluene	120	BQ (1.0) DMSO (4.0)	Ag_2_CO_3_ (2.0) H_2_O (8.0)	61
3^[d]^	DCM	135	BQ (1.0) DMSO (4.0)	Ag_2_CO_3_ (2.0) H_2_O (8.0)	60
4^[d]^	DCB	140	BQ (1.0) DMSO (4.0)	Ag_2_CO_3_ (2.0) H_2_O (8.0)	42 (40)
5^[d]^	DCE	120	BQ (1.0) DMSO (4.0)	Ag_2_CO_3_ (2.0) H_2_O (8.0)	100 (62)
6^[d]^	toluene	120	BQ (1.0) DMSO (4.0)	Ag_2_CO_3_ (2.0) H_2_O (8.0)	100 (67)
7^[d]^	toluene	120	BQ (1.0)	Ag_2_CO_3_ (2.0)	78 (24)
8^[d]^	toluene	120	Cu(OTf)_2_ (1.0)	Ag_2_O (1.0)	44
9^[d]^	toluene	120	Cu(OTf)_2_ (1.0)	Ag_2_O (1.0)	58
10^[d]^	toluene	120	BQ (0.5)	Ag_2_O (1.0)	47 (10)
11^[d]^	THF	60	BQ (0.5)	Ag_2_O (1.0)	54
12^[d]^	THF	60	BQ (0.5)	Ag_2_O (1.0)	82
13^[d]^	THF	80	BQ (0.5)	Ag_2_O (1.0)	100 (69)
14^[d]^	THF	80	Ag_2_O (1.0)		n.c.^[e]^
15^[d]^	THF	80	BQ (0.5)		30
16^[d]^	THF	80	BQ (1.0)		59

[a] Determined by GC–MS with dodecane as the internal standard. [b] Isolated yield in parentheses. [c] 2.5 equiv. Ph—BF_3_K as aryl donor. [d] 3.0 equiv. Ph—B(OH)_2_ as aryl donor. [e] n.c.=No conversion.

This prompted us to change the aryl donor to phenylboronic acid, which led to significantly better results. If DCM was used as the solvent at 135 °C (autoclave), an increased conversion of 60 % was detected (entry 3); DCB also produced the desired product if the boronic acid was used as an aryl donor and 40 % of product was isolated (entry 4). However, biphenyl was a major byproduct and, hence, an excess of phenylboronic acid had to be used. On switching to dichloroethane, a full conversion was obtained for the first time (entry 5), and the product was isolated in 62 % yield. Full conversion was also obtained with toluene and the product was isolated in 67 % yield (entry 6). In this experiment, traces of bisarylated product were also detected according to GC–MS, but could not be isolated. If no water or DMSO were added, the conversion and isolated yields dropped significantly (entry 7).

Subsequently, we also investigated the conditions reported for the arylation of 2-phenoxypyrimidines. Using the same conditions, we found that it led to only 44 % conversion (entry 8), which improved if the 3 equivalents of boronic acid were added in several portions (58 %, entry 9). Substituting Cu(OTf)_2_ for the cheaper BQ, had no dramatic effect (entry 10). One remaining drawback of the conditions investigated thus far was that, according to TLC, the reactions were not very clean and several spots were always present, which complicated isolation by column chromatography. Based on the literature, THF was reported as a suitable solvent for pyridine-directed alkylation reactions at sp^2^ centers.^[23a]^ Indeed, this led to a reaction that was much cleaner. The initial experiment at 60 °C gave 54 % conversion (entry 11), which could be improved to 82 % if the boronic acid was added in portions (entry 12). By increasing the temperature to 80 °C, full conversion was obtained and the product was isolated in 69 % yield (entry 13). Finally, we tested whether a combination of Ag_2_O and BQ was required to achieve full conversion. Using solely Ag_2_O gave no product formation at all (entry 14). However, 30 % conversion to the desired product was observed in the absence of Ag_2_O with 0.5 equiv. of BQ (entry 15). If the amount of BQ was increased (1.0 equiv.) 59 % conversion were detected. In both cases, significant biphenyl formation was observed, which was a typical result (entry 16). A further increase in BQ led to the predominant formation of the bisarylated product (not shown).

Based on the above summarized optimization results, we extended the following conditions for substrate scope investigations: 3 equiv. of boronic acid, 10 mol % Pd(OAc)_2_, 0.5 equiv. 1,4-benzoquinone (BQ), and 1 equiv. Ag_2_O in dry THF as solvent. As biphenyl formation could not be suppressed under an argon atmosphere, reactions were conducted in air for greater operational simplicity. Adding the arylboronic acid in serial portions suppressed the biphenyl formation to some extent and, hence, a periodic administration (1 equivalent every 6 h) was employed.

Initially, *N*-phenylpyridin-2-amine was arylated by using different boronic acids (Table 2). It was found that boronic acids carrying electron-donating (entries 2 and 3) and electron-withdrawing substituents (entries 4 and 5) were well-tolerated, giving yields between 62 % and 74 %. Actually, the most electron-deficient boronic acid (3-nitrophenylboronic acid) gave the highest yield (entry 5). Then, we investigated the influence of the electronic nature of the substrate. If the aryl ring for arylation was electron-rich, such as in *N*-(4-*m*ethoxyphenyl)pyridin-2-amine, improved yields were generally found (entries 6–10). This could be attributed to a facilitated oxidative addition, owing to the higher electron density of the phenyl ring. Introduction of the phenyl group gave 83 % yield (entry 6, cf. entry 1: 69 %), *p*-tolyl 76 % (entry 7, cf. entry 2: 63 %), and 3-nitrophenyl 88 % (entry 8, cf. entry 5: 74 %). 4-acetylphenylboronic acid and 4-fluorophenylboronic acid also produced good yields (entries 9 and 10).

**Table 2:** Substrate scope of the pyridine directed direct arylation of compounds 11.
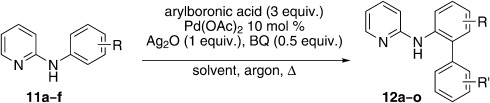

Entry^[a]^	Substrate	Boronic acid	Product	Yield [%]
1	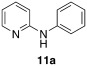	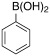	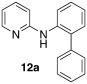	69
2	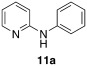	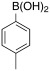	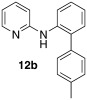	63
3	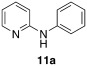	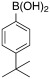	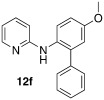	65
4	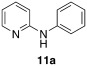	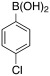	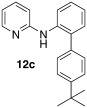	62
5	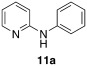	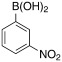	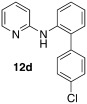	74
6	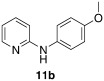	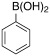	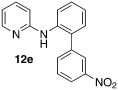	83
7	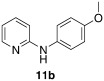	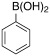	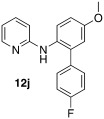	76
8	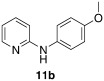	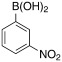	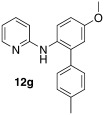	88
9	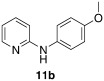	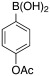	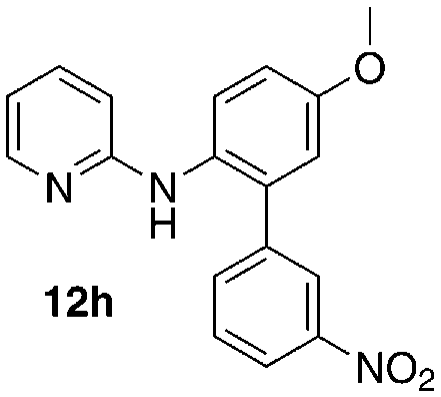	57
10	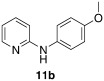	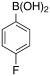	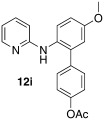	77
11	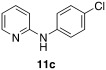	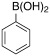	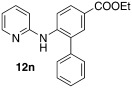	63
12	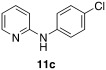	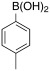	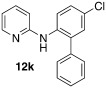	57
13	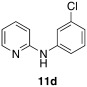	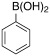	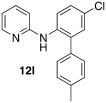	45
14	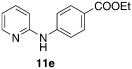	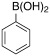	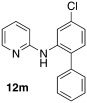	43
15	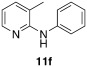	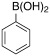	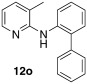	50^[a]^

[a] Bisarylated product **13** formed as a byproduct.

Switching to starting materials with electron-withdrawing substituents gave different results. In the case of *N*-(4-*c*hlorophenyl)pyridine-2-amine, yields were slightly lower compared to the unsubstituted substrate (entries 11 and 12). This effect was even more pronounced if the chlorine was located at position 3 of the phenyl ring of the starting material (entry 13). In this case, two different products could be formed, owing to the presence of two different *ortho*-positions. However, only arylation of the sterically less demanding position was observed. Also carboxylic ester functionality in the starting material led to a decreased yield of 43 % (entry 14), which was the lowest in the whole series, but in our opinion still synthetically useful. In these substrates the decreased electron density of the phenyl ring was, of course, detrimental for oxidative addition and, hence, lower reactivity and lower yields were observed. Notably, the reaction was ineffective if sterically demanding *o*-tolylboronic acid was used and had a negligible conversion. This was true for substrate **11 a** and the usually more reactive substrate **11 b**.

**Scheme 3:**
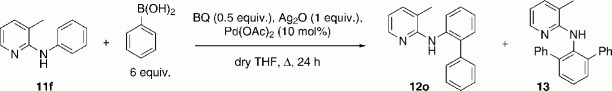
Bisarylation of substrate **11 f**.

Using starting material **11 f**, significant amounts of bisarylated product **13** were observed, which was not the case in any of the previous examples (Scheme 3). The formation of the bisarylated product can be explained by sterics. After monoarylation, the methyl group of the pyridine-directing group and the newly introduced phenyl ring incline to arrange away from each other, exposing the second *ortho* position to the directing group facilitating a second arylation reaction. By using 6 equiv. of boronic acid (in 1 equiv. portions over 24 h), mono- and bisarylated products were formed in a ratio of approximately 1:1. The isolated yield of the monoarylated product was 45 %, whereas 46 % of the bisarylated product was obtained.

### Mechanistic proposal

**Scheme 4:**
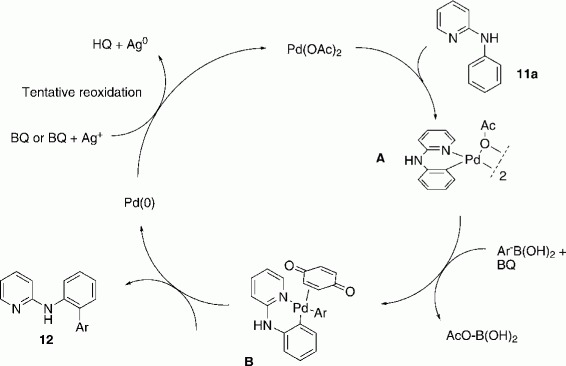
Proposed mechanism for the direct arylation of substrates **11**.

The mechanism proposed in Scheme 4 is supported by recent literature findings.^21^ The pyridine nitrogen initially coordinates the metal catalyst and facilitates insertion into the aryl C—H bond in the *ortho* position of the amino group, likely giving rise to an intermediate Pd^II^ complex such as **A**. The boronic acid then undergoes transmetalation with this complex to form AcO—B(OH)_2_ and **B**, still a Pd^II^ complex. Finally, reductive elimination from **B** delivers the product and a Pd^0^ species which is reoxidized by BQ or a combination of BQ and Ag_2_O to Pd^II^ and then re-enters the catalytic cycle.

It was stated in the literature that BQ is required for the C—H activation and reductive elimination step^23^ and for reoxidation of Pd^0^ to Pd^II^.10b If BQ alone was needed for the reoxidation of Pd^0^ to Pd^II^, at least some conversion (maximum 10 % for one full turnover of catalyst) should have been observed in its absence. However, our findings did not confirm this (Table 1, entry 14). Hence, BQ might also have played an important role as a ligand.^21^, 9a Several literature reports state that BQ promotes reoxidation of Pd^0^ to Pd^II^ in combination with a silver salt.9f, 24 Experiments in the absence of Ag_2_O suggested that the silver salt is actually not mandatory, although it facilitates this oxidation step (Table 1, entries 15 and 16). We found that 0.5 and 1.0 equiv. of BQ led to 30 and 59 % conversion, respectively. The oxidation potential of BQ was high enough to reoxidize Pd^0^, also in the absence of silver, but a combination of these two reagents worked significantly better. Additionally, it was reported that silver salts promoted the transmetalation step, which might also be the case in our transformation.9f Interestingly, we found that only parts of BQ were reduced to hydroquinone, as BQ could still be detected by GC–MS after full conversion.

**Scheme 5:**
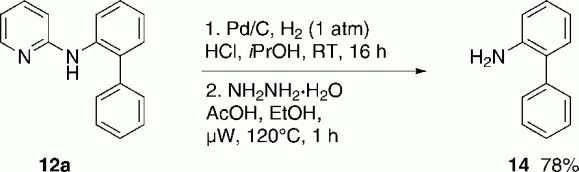
Cleavage of the pyridyl directing group.

Finally, we showed that the pyridine-directing group could be cleaved according to a literature-known procedure in two steps (Scheme 5).^25^ Pd-catalyzed hydrogenation and subsequent treatment with NH_2_NH_2_ and HCl furnished 2-aminobiphenyl **14** in good yield (78 %). This demonstrated that pyridine could be considered as the removable directing group and 2-aminobiphenyls were formed from this reaction sequence. These are important ligands for metal catalysts^26^ and structural motifs in organic electroluminescent devices.^27^

## Conclusions

An efficient method has been developed for arylation in the *ortho* position of anilines directed by pyridine. The reaction is robust regarding electronic effects because the electron rich substrates are more reactive. Regarding the aryl donors, electron poor boronic acids give higher yields compared to their electron rich counterparts. Sterically demanding boronic acids were not tolerated. Introducing steric bulk in the pyridine-directing group of the substrate leads to the bisarylation product. The reaction does not require inert conditions and is operationally simple. A mild temperature of 80 °C can be used and boronic acids act as readily available aryl donors. Cleavage of the pyridine directing group is also demonstrated and gives a good yield of 78 % over two steps. A plausible mechanism is proposed based on our observations.

After completion of our optimization experiments and substrate scope investigations, Wu and co-workers published a procedure in which *N*-2-pyridyl-carbazoles were formed from the type of starting materials used here.^21^ Also in this transformation Pd(OAc)_2_, a silver salt (AgOAc) and BQ were used together with Ar—BF_3_K salts as the aryl donor. The silver salt in a threefold excess and a full equivalent of BQ were required. 1,4-Dioxane and 4 equiv. DMSO were identified as the most effective solvents. Eventually, by increasing the amounts of oxidants and by using our boronic acid method disclosed in this contribution, it could be possible to form carbazoles as well. We intend to investigate this in the near future.

## Experimental Section

Unless otherwise noted, chemicals were purchased from commercial suppliers and used without further purification. Microwave reactions were performed on a Biotage Initiator Sixty microwave unit. Flash column chromatography was performed on silica gel 60 from Merck (40–63 μm), whereas most separations were performed by using a Büchi SepacoreTM MPLC system with a 45 g column. For TLC aluminum-backed silica gel was used. Melting points were determined by using a Kofler-type Leica Galen III micro hot stage microscope and are uncorrected. HR-MS for compounds unknown in the literature were performed by E. Rosenberg at Vienna University of Technology, Institute for Chemical Technologies and Analytics; all samples were analyzed by LC-IT-TOF-MS in only positive ion detection mode with the recording of MS and MS/MS spectra. NMR-spectra were recorded in CDCl_3_ with TMS as internal standard on a Bruker AC 200 (200 MHz) spectrometer and chemical shifts are reported in ppm. For assignment of ^13^C multiplicities standard ^13^C and DEPT spectra were recorded. GC–MS runs were performed on a Thermo Finnigan Focus GC/DSQ II with a standard capillary column BGB 5 (*ID*=30 m×0.32 mm).

### General method A: Preparation of starting materials

2-Bromopyridine (1 equiv.), amine (1.2 equiv.), NaO*t*Bu (2.0 equiv.) or K_2_CO_3_ (10 equiv., in case of **1 d**), Pd(OAc)_2_ (2 mol %) and (+/−)-BINAP (2 mol %) were taken in a closed vial and the reaction vessel was flushed with argon. Dry toluene was added to it through the septum and then it was placed in a heating block at 120 °C overnight. Purification by column chromatography was performed by using light petroleum (LP)/EtOAc to obtain the desired starting material **11 a**,^28^ **11 b**,^28^ **11 c**,^29^ **11 d**,^29^ **11 e**,^28^ or **11 f**,^30^ in excellent yield.

### General method B: Direct arylation

*N*-aryl-2-aminophenyl pyridine (1 equiv.), aryl boronic acid (total 3 equiv. in 1. equiv. portions), BQ (0.5 equiv.), Ag_2_O (1 equiv.), and Pd(OAc)_2_ (10 mol %) were placed in a screw-cap vial with dry THF (4 mL) and the reaction mixture was stirred on a heating block at 80 °C for 24 h. A portion of aryl boronic acid (1 equiv.), was added at the start, followed by a portion (1 equiv.) after both 6 and 12 h. Reactions were monitor by TLC and GC—MS. Purification by column chromatography was performed by using LP/EtOAc. The following compounds were prepared using this method.

*N*-([1,1′-biphenyl]-2-yl)pyridin-2-amine (**12 a**): Substrate **11 a** (50 mg, 0.29 mmol), phenylboronic acid (35 mg, 0.29 mmol), Ag_2_O (67 mg, 0.29 mmol), BQ (16 mg, 0.15 mmol), Pd(OAc)_2_ (6.5 mg, 0.029 mmol). Column chromatography 10:1 LP/EtOAc. Yield: 69 % (50 mg, 0.20 mmol) yellow oil. *R*_f_=0.26 (LP/EtOAc=10:1). GC–MS: 246 (69, *M*^+^), 245 (100), 169 (64), 230 (29), 167 (13). *δ*=6.46 (s, 1 H), 6.64–6.74 (m, 1 H), 6.79 (d, *J*=8.4 Hz, 1 H), 7.07–7.17 (m, 1 H), 7.23–7.51 (m, 8 H), 7.76 ppm (d, *J*=8.2 Hz, 1 H). ^13^C NMR (CDCl_3_, 50 MHz): *δ*=108.6 (d), 115.1 (d), 120.6 (d), 122.9 (d), 127.6 (d), 128.2 (d), 128.8 (d), 129.3 (d), 130.8 (d), 133.3 (s), 137.4 (s), 137.6 (d), 138.8 (s), 148.4 (d), 155.9 ppm (s). HR-MS: Predicted [*M*H]^+^=247.1230; Measured [*M*H]^+^=247.1220 (diff. in ppm=−4.05).

*N*-(4′-methyl-[1,1′-biphenyl]-2-yl)pyridin-2-amine (**12 b**): Substrate **11 a** (50 mg, 0.29 mmol), 4-methylphenylboronic acid (39 mg, 0.29 mmol), Ag_2_O (67 mg, 0.29 mmol), BQ (16 mg, 0.15 mmol), Pd(OAc)_2_ (6.5 mg, 0.029 mmol). Column chromatography 10:1 LP/EtOAc. Yield: 63 % (48 mg, 0.18 mmol) yellow oil. *R*_f_=0.25 (LP/EtOAc=10:1). GC–MS: 260 (78, *M*^+^), 259 (100), 244 (40), 169 (54), 129 (20). ^1^H NMR (CDCl_3_, 200 MHz): *δ*=2.39 (s, 3 H), 6.45 (s, 1 H), 6.65–6.74 (m, 1 H), 7.05–7.15 (m, 1 H), 7.17–7.34 (m, 6 H), 7.35–7.57 ppm (m, 1 H). ^13^C NMR (CDCl_3_, 50 MHz): *δ*=21.2 (q), 108.6 (d), 115.0 (d), 120.6 (d), 122.0 (d), 129.7 (d), 129.6 (d), 130.8 (d), 133.4 (s), 135.8 (s), 137.3 (s), 137.5 (s), 137.7 (d), 148.3 (d), 156.0 ppm (s). HR-MS: Predicted [*M*H]^+^=261.1386; Measured [*M*H]^+^=261.1374 (diff. in ppm=−4.60).

*N*-(4′-(*tert*-butyl)-[1,1′-biphenyl]-2-yl)pyridin-2-amine (**12 c**): Substrate **11 a** (50 mg, 0.29 mmol), 4-*tert*-butylphenylboronic acid (52 mg, 0.29 mmol), Ag_2_O (67 mg, 0.29 mmol), BQ (16 mg, 0.15 mmol), Pd(OAc)_2_ (6.5 mg, 0.029 mmol). Column chromatography 10:1 LP/EtOAc. Yield: 65 % (63 mg, 0.22 mmol) yellow oil. *R*_f_=0.47 (LP/EtOAc=10:1). GC–MS: 302 (60, *M*^+^), 301 (51), 286 (40), 169 (100), 129 (51). ^1^H NMR (CDCl_3_, 200 MHz): *δ*=1.10 (s, 9 H), 6.20 (s, 1 H), 6.46 (dd, *J*_1_=7.0 Hz, *J*_2_=1.6 Hz, 1 H), 6.62 (d, *J*=8.4 Hz, 1 H), 6.79–6.90 (m, 1 H), 7.01–7.12 (m, 1 H), 7.14–7.27 (m, 3 H), 7.52 (d, *J*=8.0 Hz, 1 H), 7.92 ppm (dd, *J*_1_=4.9 Hz, *J*_2_=1.4 Hz, 1 H). ^13^C NMR (CDCl_3_, 50 MHz): *δ*=31.3 (q), 34.6 (s), 108.7 (d), 115.0 (d), 120.4 (d), 125.8 (d), 129.0 (d), 130.9 (d), 133.2 (s), 135.7 (s), 137.5 (s), 137.7 (s), 148.4 (d), 150.5 (s), 155.9 ppm (s). HR-MS: Predicted [*M*H]^+^=303.1862; Measured [*M*H]^+^=303.1856 (diff. in ppm=1.98).

*N*-(4′-chloro-[1,1′-biphenyl]-2-yl)pyridin-2-amine (**12 d**): Substrate **11 a** (50 mg, 0.29 mmol), 4-chlorophenylboronic acid (45 mg, 0.29 mmol), Ag_2_O (67 mg, 0.29 mmol), BQ (16 mg, 0.15 mmol), Pd(OAc)_2_ (6.5 mg, 0.029 mmol). Column chromatography 10:1 LP/EtOAc. Yield: 62 % (51 mg, 0.18 mmol) yellow oil. *R*_f_=0.49 (LP/EtOAc=10:1). GC–MS: 281 (32, *M*^+^), 280 (36), 264 (25), 143 (21), 169 (100). ^1^H NMR (CDCl_3_, 200 MHz): *δ*=6.33 (s, 1 H), 6.66 −6.81 ( m, 2 H), 7.07–7.18 (m, 7 H), 7.74 (d, *J*=7.8 Hz, 1 H), 8.10–8.19 ppm (m, 1 H). ^13^C NMR (CDCl_3_, 50 MHz): *δ*=108.4 (d), 115.2 (d), 121.3 (d), 123.4 (d), 128.6 (d), 129.0 (d), 130.6 (d), 132.5 (s), 133.6 (s), 137.3 (s), 137.4 (s), 137.7 (d), 148.4 (d), 155.8 ppm (s). HR-MS: Predicted [*M*H]^+^=281.0840; Measured [*M*H]^+^=281.0843 (diff. in ppm=1.07).

*N*-(3′-nitro-[1,1′-biphenyl]-2-yl)pyridin-2-amine (**12 e**): Substrate **11 a** (50 mg, 0.29 mmol), 3-nitrophenylboronic acid (48 mg, 0.29 mmol), Ag_2_O (67 mg, 0.29 mmol), BQ (16 mg, 0.15 mmol), Pd(OAc)_2_ (6.5 mg, 0.029 mmol). Column chromatography 10:1 LP/EtOAc. Yield: 74 % (63 mg, 0.22 mmol) yellow oil. *R*_f_=0.16 (LP/EtOAc=10:1). GC–MS: 291 (34, *M*^+^), 290 (34,), 244 (32), 243 (44), 169 (100). ^1^H NMR (CDCl_3_, 200 MHz): *δ*=6.36 (s, 1 H), 7.06–7.19 (m, 1 H), 7.20–7.49 (m, 4 H), 7.55–7.72 (m, 2 H), 7.95–8.11 (m, 2 H), 8.15–8.21 ppm (m, 1 H). ^13^C NMR (CDCl_3_, 50 MHz): *δ*=108.3 (d), 115.3 (d), 122.4 (d), 122.7 (d), 124.2 (d), 129.5 (d), 129.6 (d), 130.8 9d), 132.2 (s), 135.4 (s), 137.5 (s), 137.8 (d), 140.8 (s), 148.4 (d), 148.5 (s), 155.9 ppm (s). HR-MS: Predicted [*M*H]^+^=292.1081; Measured [*M*H]^+^=292.1081 (diff. in ppm=0).

*N*-(5-methoxy-[1,1′-biphenyl]-2-yl)pyridin-2-amine (**12 f**): Substrate **11 b** (50 mg, 0.25 mmol), phenylboronic acid (30.5 mg, 0.25 mmol), Ag_2_O (57 mg, 0.25 mmol), BQ (14 mg, 0.13 mmol), Pd(OAc)_2_ (5.6 mg, 0.025 mmol). Column chromatography 10:1 LP/EtOAc. Yield: 83 % (57 mg, 0.21 mmol) yellow oil. *R*_f_=0.32 (LP/EtOAc=5:1). GC–MS: 276 (100, *M*^+^), 275 (51), 261 (42), 199 (52), 116 (25). ^1^H NMR (CDCl_3_, 200 MHz): *δ*=3.84 (s, 3 H), 6.21 (s, 1 H), 6.57–6.70 (m, 2 H), 6.86–6.98 (m, 2 H), 7.27–7.48 (m, 6 H), 7.50–7.61 (m, 1 H), 8.05–8.15 ppm (m, 1 H). ^13^C NMR (CDCl_3_, 50 MHz): *δ*=55.6 (q), 107.5 (d), 113.8 (d), 114.2 (d), 115.8 (d), 125.1 (d), 127.6 (d), 128.6 (d), 129.9 (d), 130.2 (d), 136.8 (s), 137.6 (d), 138.8 (s), 148.2 (d), 156.3 (s), 157.1 ppm (s). HR-MS: Predicted [*M*H]^+^=277.1335; Measured [*M*H]^+^=277.1331 (diff. in ppm=−1.44).

*N*-(5-methoxy-4′-methyl-[1,1′-biphenyl]-2-yl)pyridin-2-amine (**12 g**): Substrate **11 b** (50 mg, 0.25 mmol), 4-methyl boronic acid (34 mg, 0.25 mmol), Ag_2_O (57 mg, 0.25 mmol), BQ (14 mg, 0.13 mmol), Pd(OAc)_2_ (5.6 mg, 0.025 mmol). Column chromatography 10:1 LP/EtOAc. Yield: 76 % (55 mg, 0.19 mmol) yellow oil. *R*_f_=0.18 (LP/EtOAc=10:1). GC–MS: 290 (100, *M*^+^), 289 (51), 275 (39), 199 (42), 78 (43). ^1^H NMR (CDCl_3_, 200 MHz): *δ*=2.40 (s, 3 H), 3.82 (s, 3 H), 6.17 (s, 1 H), 6.58 −6.69 (m, 2 H), 6.84–6.96 (m, 2 H), 7.15–7.30 (m, 4 H), 7.35–7.47 (m, 1 H), 7.50–7.60 (m, 1 H), 8.05–8.14 ppm (m, 1 H). ^13^C NMR (CDCl_3_, 50 MHz): *δ*=21.1 (q), 55.6 (q), 107.4 (q), 113.6 (d), 114.2 (d), 115.8 (d), 124.9 (d), 128.9 (d), 129.4 (d), 130.3 (s), 135.8 (s), 136.8 (s), 137.4 (d), 148.2 (d), 157.2 (s), 156.2 ppm (s). HR-MS: Predicted [*M*H]^+^=291.1492; Measured [*M*H]^+^=291.1491 (diff. in ppm=−0.34).

*N*-(5-methoxy-3′-nitro-[1,1′-biphenyl]-2-yl)pyridin-2-amine (**12 h**): Substrate **11 b** (50 mg, 0.25 mmol), 3-nitrophenylboronic acid (42 mg, 0.25 mmol), Ag_2_O (57 mg, 0.25 mmol), BQ (14 mg, 0.13 mmol), Pd(OAc)_2_ (5.6 mg, 0.025 mmol). Column chromatography 10:1 LP/EtOAc. Yield: 88 % (71 mg, 0.22 mmol) yellow oil. *R*_f_=0.18 (LP/EtOAc=10:1). GC–MS: 321 (100, *M*^+^), 320 (36), 306 (29), 207 (28), 199 (72). ^1^H NMR (CD_3_OD, 200 MHz): *δ*=3.84 (s, 3 H), 6.34–6.47 (m, 1 H), 6.49–6.66 (m, 3 H), 6.91–7.10 (m, 2 H), 7.27–7.41 (m, 2 H), 7.43–7.56 (m, 1 H), 7.73–7.86 (m, 2 H), 8.01–8.12 (m, 1 H), 8.21–8.32 ppm (m, 1 H). ^13^C NMR (CD_3_OD, 50 MHz): *δ*=56.1 (q), 109.7 (d), 114.7 (d), 116.8 (d), 122.9 (d), 124.8 (d), 130.4 (d), 131.3 (s), 136.4 (d), 138.8 (s), 139.2 (d), 142.8 (s), 151.3 (s), 159.4 ppm (s). HR-MS: Predicted [*M*H]^+^=322.1186; Measured [*M*H]^+^=322.1194 (diff. in ppm=2.84).

5′-Methoxy-2′-(pyridin-2-ylamino)-[1,1′-biphenyl]-4-yl acetate (**12 i**): Substrate **11 b** (50 mg, 0.25 mmol), 4-acetylphenylboronic acid (41 mg, 0.25 mmol), Ag_2_O (57 mg, 0.25 mmol), BQ (14 mg, 0.13 mmol), Pd(OAc)_2_ (5.6 mg, 0.025 mmol). Column chromatography 5:1 LP/EtOAc, followed by a preparative TLC by using LP/EtOAc=3:1 and 1 % NEt_3_. Yield: 57 % (45 mg, 0.18 mmol) yellow oil. *R*_f_=0.15 (LP/EtOAc=5:1). GC–MS: 318 (100), 317 (50), 303 (38), 199 (66), 78 (42). ^1^H NMR (CDCl_3_, 200 MHz): *δ*=2.60 (s, 3 H), 3.84 (s, 3 H), 6.10 (s, 1 H), 6.53–6.69 (m, 2 H), 6.87–7.01 (m, 2 H), 7.35–7.60 (m, 4 H), 7.95 (d, *J*=8.2 Hz, 2 H), 8.05 ppm (d, *J*=4.9 Hz, 1 H). ^13^C NMR (CDCl_3_, 50 MHz): *δ*=26.6 (q), 55.6 (q), 107.2 (d), 114.4 (d), 114.5 (d), 115.7 (d), 126.1 (d), 128.6 (d), 129.3 (d), 130.1 (s), 136.0 (s), 136.2 (s), 137.7 (d), 143.9 (s), 148.4 (d), 156.6 (s), 157.2 (s), 197.7 ppm (s). HR-MS: Predicted [*M*H]^+^=319.1441; Measured [*M*H]^+^=319.1450 (diff. in ppm=2.82).

*N*-(4′-fluoro-5-methoxy-[1,1′-biphenyl]-2-yl)pyridin-2-amine (**12 j**): Substrate **11 b** (50 mg, 0.25 mmol), 4-fluorophenylboronic acid (35 mg, 0.25 mmol), Ag_2_O (57 mg, 0.25 mmol), BQ (14 mg, 0.13 mmol), Pd(OAc)_2_ (5.6 mg, 0.025 mmol). Column chromatography 10:1 LP/EtOAc. Yield: 77 % (36.7 mg, 0.19 mmol) yellow oil. *R*_f_=0.16 (LP/EtOAc=10:1). GC–MS: 294 (100, *M*^+^), 293 (55), 279 (42), 199 (51), 78 (61). ^1^H NMR (CDCl_3_, 200 MHz): *δ*=3.84 (s, 3 H), 6.04 (s, 1 H), 6.54–6.71 (m, 2 H), 6.84–6.98 (m, 2 H), 6.98 −7.46 (m, 3 H), 7.52 (d, *J*=8.6 Hz, 1 H), 8.10 ppm (d, *J*=3.5 Hz, 1 H). ^13^C NMR (CDCl_3_, 50 MHz): *δ*=55.6 (t), 107.2 (d), 113.9 (d), 114.3 (d), 115.6 (d, *J*_CF_=21.4 Hz), 115.8 (d), 125.6 (d), 130.2 (s), 130.7 (d, *J*_CF_=8.0 Hz), 134.8 (s, *J*_CF_=3.4 Hz), 136.2 (s), 137.6 (d), 148.4 (d), 156.4 (s), 157.2 (s), 162.3 ppm (d, *J*_CF_=247 Hz). HR-MS: Predicted [*M*H]^+^=295.1241; Measured [*M*H]^+^=295.1241 (diff. in ppm=0.0).

*N*-(5-chloro-[1,1′-biphenyl]-2-yl)pyridin-2-amine **(12 k**): Substrate **11 c** (50 mg, 0.24 mmol), phenylboronic acid (29 mg, 0.24 mmol), Ag_2_O (55 mg, 0.24 mmol), BQ (13 mg, 0.12 mmol), Pd(OAc)_2_ (5.4 mg, 0.024 mmol). Column chromatography 10:1 LP/EtOAc. Yield: 63 % (43 mg, 0.15 mmol) yellow oil. *R*_f_=0.26 (LP/EtOAc=10:1). GC–MS: 281 (30, *M*^+^), 280 (51), 264 (23), 143 (19), 169 (100). ^1^H NMR (CDCl_3_, 200 MHz): *δ*=6.25–6.41 (s, 1 H), 6.63–6.80 (m, 2 H), 7.22–7.33 (m, 2 H), 7.34–7.53 (m, 6 H), 7.86 (d, *J*=8.2 Hz, 1 H), 8.12–8.22 ppm (m, 1 H). ^13^C NMR (CDCl_3_, 50 MHz): *δ*=109.1 (d), 115.4 (d), 121.6 (d), 127.4 (s), 128.0 (d), 128.1 (d), 129.0 (d), 129.2 (d), 130.3 (d), 134.4 (s), 136.2 (s), 137.5 (s), 137.7 (d), 148.3 (d), 155.5 ppm (s). HR-MS: Predicted [*M*H]^+^=281.0840; Measured [*M*H]^+^=281.0844 (diff. in ppm=1.42).

*N*-(5-chloro-4′-methyl-[1,1′-biphenyl]-2-yl)pyridin-2-amine (**12 l)**: Substrate **11 c** (50 mg, 0.24 mmol), 4-methylphenylboronic acid (34 mg, 0.24 mmol), Ag_2_O (55 mg, 0.24 mmol), BQ (13 mg, 0.12 mmol), Pd(OAc)_2_ (5.4 mg, 0.024 mmol). Column chromatography 10:1 LP/EtOAc. Yield: 57 % (41 mg, 0.14 mmol) yellow oil. *R*_f_=0.43 (LP/EtOAc=10:1). GC–MS: 296 (33), 295 (44, *M*^+^), 294 (100), 193 (89), 278 (51). ^1^H NMR (CDCl_3_, 200 MHz): *δ*=2.39 (s, 3 H), 6.37 (s, 1 H), 6.66–6.78 (m, 2 H), 7.19–7.32 (m, 6 H), 7.41–7.52 (m, 1 H), 7.80–7.89 (m, 1 H), 8.13–8.21 ppm (m, 1 H). ^13^C NMR (CDCl_3_, 50 MHz): *δ*=109.1 (d), 115.3 (d), 121.5 (d), 127.4 (s), 129.0 (d), 129.7 (d), 130.3 (d), 134.5 (s), 136.2 (s), 137.7 (d), 137.9 (s), 148.2 (d), 155.5 ppm (s). HR-MS: Predicted [*M*H]^+^=295.0097; Measured [*M*H]^+^=295.1003 (diff. in ppm=2.03).

*N*-(4-chloro-[1,1′-biphenyl]-2-yl)pyridin-2-amine (**12 m**): Substrate **11 d** (50 mg, 0.24 mmol), phenylboronic acid (29 mg, 0.24 mmol), Ag_2_O (55 mg, 0.24 mmol), BQ (13 mg, 0.12 mmol), Pd(OAc)_2_ (5.4 mg, 0.024 mmol). Column chromatography 10:1 LP/EtOAc. Yield: 45 % (31 mg, 0.11 mmol) yellow oil. *R*_f_=0.37 (LP/EtOAc=10:1). GC–MS: 281 (30, *M*^+^), 280 (60), 279 (69), 203 (100), 121 (34). ^1^H NMR (CDCl_3_, 200 MHz): *δ*=6.47 (s, 1 H), 6.69–6.83 (m, 2 H), 7.00–7.10 (m, 1 H), 7.15–7.29 (m, 2 H), 7.33–7.58 (m, 6 H), 8.03 (d, *J*=2.5 Hz, 1 H), 8.16–8.26 ppm (m, 1 H). ^13^C NMR (CDCl_3_, 50 MHz): *δ*=109.6 (d), 115.7 (d), 119.3 (d), 122.3 (d), 127.9 (d), 129.0 (d), 129.3 (d), 130.6 (s), 131.5 (d), 133.8 (s), 137.7 (s), 137.8 (d), 138.7 (s), 148.1 (d), 154.9 ppm (s). HR-MS: Predicted [*M*H]^+^=281.0840; Measured [*M*H]^+^=281.0845 (diff. in ppm=1.78).

Ethyl 6-(pyridin-2-ylamino)-[1,1′-biphenyl]-3-carboxylate (**12 n**): Substrate **11 e** (50 mg, 0.21 mmol), phenylboronic acid (25.6 mg, 0.21 mmol), Ag_2_O (48 mg, 0.21 mmol), BQ (12 mg, 0.11 mmol), Pd(OAc)_2_ (4.7 mg, 0.021 mmol). Column chromatography 10:1 LP/EtOAc. Yield: 43 % (28 mg, 0.09 mmol) yellow oil. *R*_f_=0.17 (LP/EtOAc=10:1). GC–MS: 318 (100, *M*^+^), 317 (81), 302 (24), 289 (39), 241 (51). ^1^H NMR (CDCl_3_, 200 MHz): *δ*=1.38 (t, *J*=7.2 Hz, 3 H), 4.36 (q, *J*=7.2 Hz, 2 H), 6.72 (s, 1 H), 6.78–6.88 (m, 2 H), 7.35–7.59 (m, 6 H), 7.91–8.15 (m, 3 H), 8.24 ppm (d, *J*=4.5 Hz, 1 H). ^13^C NMR (CDCl_3_, 50 MHz): *δ*=14.4 (q), 60.7 (t), 110.6 (q), 116.4 (d), 117.1 (d), 123.3 (s), 128.1 (d), 129.2 (d), 129.4 (d), 130.1 (d), 130.8 (d), 132.1 (d), 137.8 (d), 142.0 (s), 148.2 (d), 154.4 (s), 166.4 ppm (s). HR-MS: Predicted [*M*H]^+^=319.1441; Measured [*M*H]^+^=319.1453 (diff. in ppm=3.76).

*N*-([1,1′-biphenyl]-2-yl)-3-methylpyridin-2-amine (**12 o**): Substrate **11 f** (50 mg, 0.27 mmol), 2-methylphenylboronic acid (33 mg, 0.27 mmol), Ag_2_O (62 mg, 0.27 mmol), BQ (16 mg, 0.15 mmol), Pd(OAc)_2_ (6.0 mg, 0.027 mmol). Column chromatography 10:1 LP/EtOAc. Yield: 50 % (35 mg, 0.13 mmol) yellow oil. *R*_f_=0.48 (LP/EtOAc=10:1). GC–MS: 260 (58, *M*^+^), 259 (100), 183 (90), 152 (14), 129 (15). ^1^H NMR (CDCl_3_, 200 MHz): *δ*=1.85 (s, 3 H), 6.36 (s, 1 H), 6.68 (dd, *J*_1_=7.2 Hz, *J*_2_=5.1 Hz, 1 H), 7.01–7.11 (m, 1 H), 7.21–7.31 (m, 2 H), 7.32–7.49 (m, 6 H), 8.06–8.15 (m, 1 H), 8.40 ppm (d, *J*=8.02 Hz, 1 H). ^13^C NMR (CDCl_3_, 50 MHz): *δ*=16.9 (q), 115.1 (d), 118.5 (s), 120.0 (d), 121.7 (d), 127.7 (d), 128.3 (d), 129.4 (d), 129.9 (d), 131.6 (s), 137.8 (d), 139.0 (s), 145.1 (d), 153.9 ppm (s). HR-MS: Predicted [*M*H]^+^=261.1386; Measured [*M*H]^+^=261.1374 (diff. in ppm=−4.60).

*N*-([1,1′:3′,1′′-terphenyl]-2′-yl)-3-methylpyridin-2-amine (**13**): **1 b** (50 mg, 0.27 mmol), 2-methylphenylboronic acid (33 mg, 0.27 mmol), Ag_2_O (62 mg, 0.27 mmol), BQ (16 mg, 0.15 mmol), Pd(OAc)_2_ (6.0 mg, 0.027 mmol). Prepared according to general procedure B, except with 6 equiv. of boronic acid. Column chromatography 10:1 LP/EtOAc, followed by preparative HPLC (*n*-heptane/*i*PrOH=90:10). Yield: 46 % (25 mg, 0.07 mmol) yellow oil. *R*_f_=0.40 (LP/EtOAc=10:1). GC–MS: 336 (13, *M*^+^), 260 (23), 259 (100), 258 (5), 257 (5). ^1^H NMR (CDCl_3_, 200 MHz): *δ*=1.82 (s, 3 H), 5.51 (s, 1 H), 6.37 (dd, *J*_1_=7.2 Hz” *J*_2_=5.1 Hz, 1 H), 6.97–7.07 (m, 1 H), 7.12–7.30 (m, 6 H), 7.31–7.44 (m, 7 H), 7.70–7.77 ppm (m, 1 H). ^13^C NMR (CDCl_3_, 50 MHz): *δ*=17.0 (t), 114.3 (d), 117.8 (s), 125.5 (d), 126.8 (d), 127.9 (d), 128.8 (d), 130.1 (d), 135.2 (s), 136.3 (d), 138.5 (s), 140.3 (s), 145.4 (d), 154.8 ppm (s). HR-MS: Predicted [*M*H]^+^=261.1386; Measured [*M*H]^+^=261.1374 (diff. in ppm=−4.60).

2-phenylaniline **14**: A 3-neck flask was charged with Pd/C (80 mg; 10 % Pd-basis) and *i*PrOH (5 mL) and the mixture was stirred for 5 min under N_2_. Afterwards, **2 a** (246 mg, 1 mmol), which was dissolved in *i*PrOH (10 mL), and 2 n HCl (3 mL, 6 mmol, 6 equiv.) were added to the solution. The resulting mixture was flushed with H_2_ three times and then stirred under H_2_ (1 atm, 101 kPa) at 50 °C overnight. Then, the solids were removed by filtration through Celite, and the solution was evaporated to dryness. Afterwards, 1 M NaOH solution (8 mL) was added, and the reaction mixture was extracted with DCM (3×10 mL). The combined organic layers were dried over Na_2_SO_4_, filtered and evaporated to dryness. The crude product (231 mg) was dissolved in 5 mL NH_2_NH_2_**⋅**H_2_O/AcOH (2.5:0.7 M in EtOH) in a microwave vial and flushed with argon. The vial was heated up to 120 °C for 1 h in microwave. The reaction mixture was allowed to cool to ambient temperature and the volatiles were removed under reduced pressure. After addition of 1 n NaOH solution (5 mL), the mixture was extracted with Et_2_O (3×10 mL). The combined organic layers were dried over Na_2_SO_4_, filtered, and evaporated to dryness. The product was dried under high vacuum to give the pure amine **14** (131 mg) in 78 % yield as a pale yellow solid. M.p.=48–49 °C; The spectral data is in agreement with literature values.^31^ ^1^H NMR (CDCl_3_, 200 MHz): *δ*=3.69 (br s, 2 H), 6.73–6.85 (m, 2 H), 7.11–7.18 (m, 2 H), 7.33–7.45 ppm (m, 5 H). ^13^C NMR (CDCl_3_, 50 MHz): *δ*=115.7 (d), 118.7 (d), 127.3 (d), 127.7 (s), 128.6 (d), 128.9 (d), 129.2 (d), 130.6 (d), 139.6 (s), 143.6 ppm (s).
