# Supplementary material for: Palladium(II)-Catalyzed Regioselective Ortho Arylation of sp2 C—H Bonds of N-Aryl-2-amino Pyridine Derivatives
Source: ChemCatChem. 2012 Jun 13;4(9):1345–52. doi: 10.1002/cctc.201200155 (PMC3483625; doi:10.1002/cctc.201200155)

Heterogeneous & Homogeneous & Bio-
CHEMCATCHEM
CATALYSIS

Supporting Information

© Copyright Wiley-VCH Verlag GmbH & Co. KGaA, 69451 Weinheim, 2012

Palladium(II)-Catalyzed Regioselective *Ortho* Arylation of sp^2 C–H Bonds of *N*-Aryl-2-amino Pyridine Derivatives

Moumita Koley, Navid Dastbaravardeh, Michael Schnürch,* and Marko D. Mihovilovic[a]

cctc_201200155_sm_miscellaneous_information.pdf

***N*-([1,1'-biphenyl]-2-yl)pyridin-2-amine (12a):**

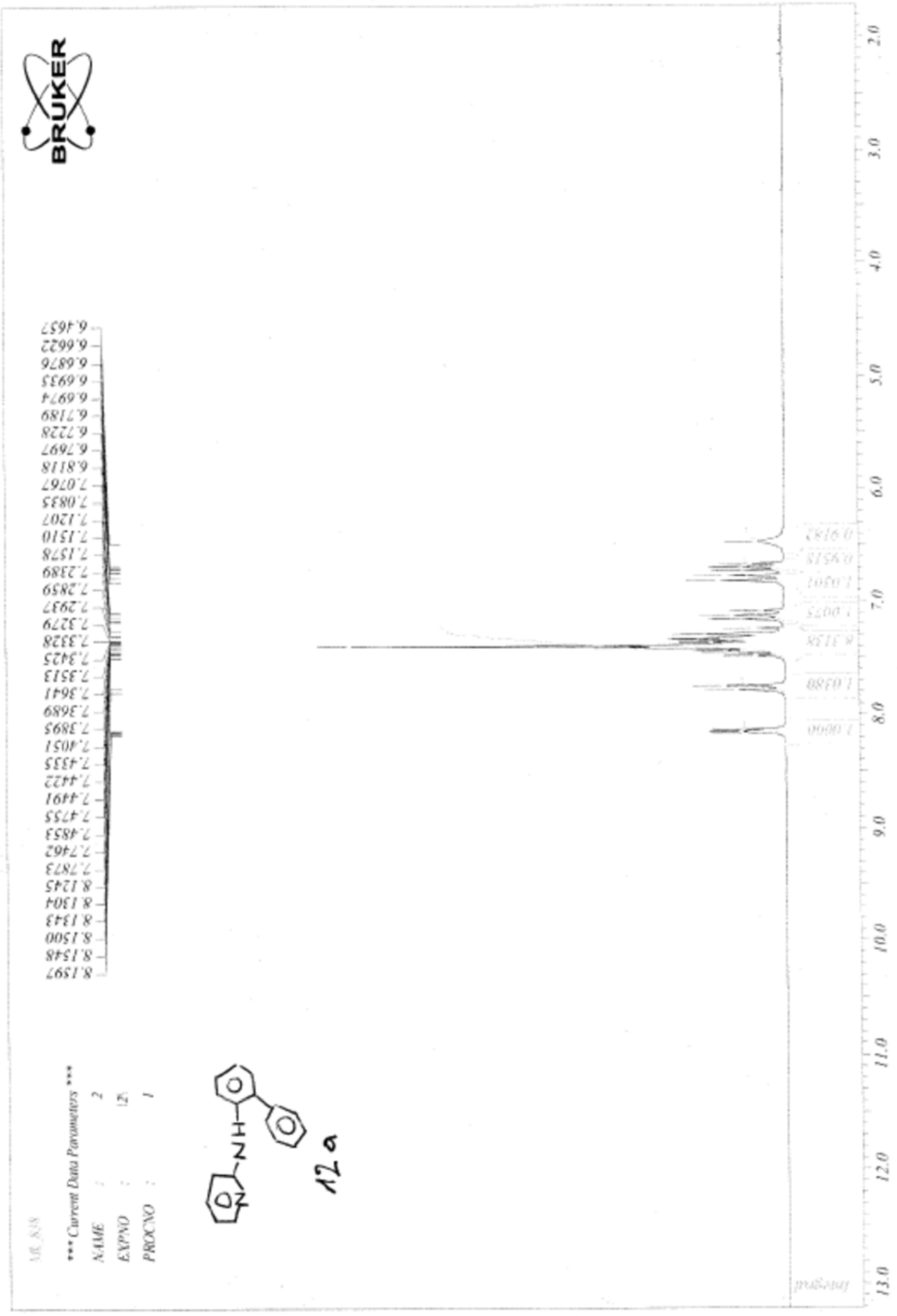

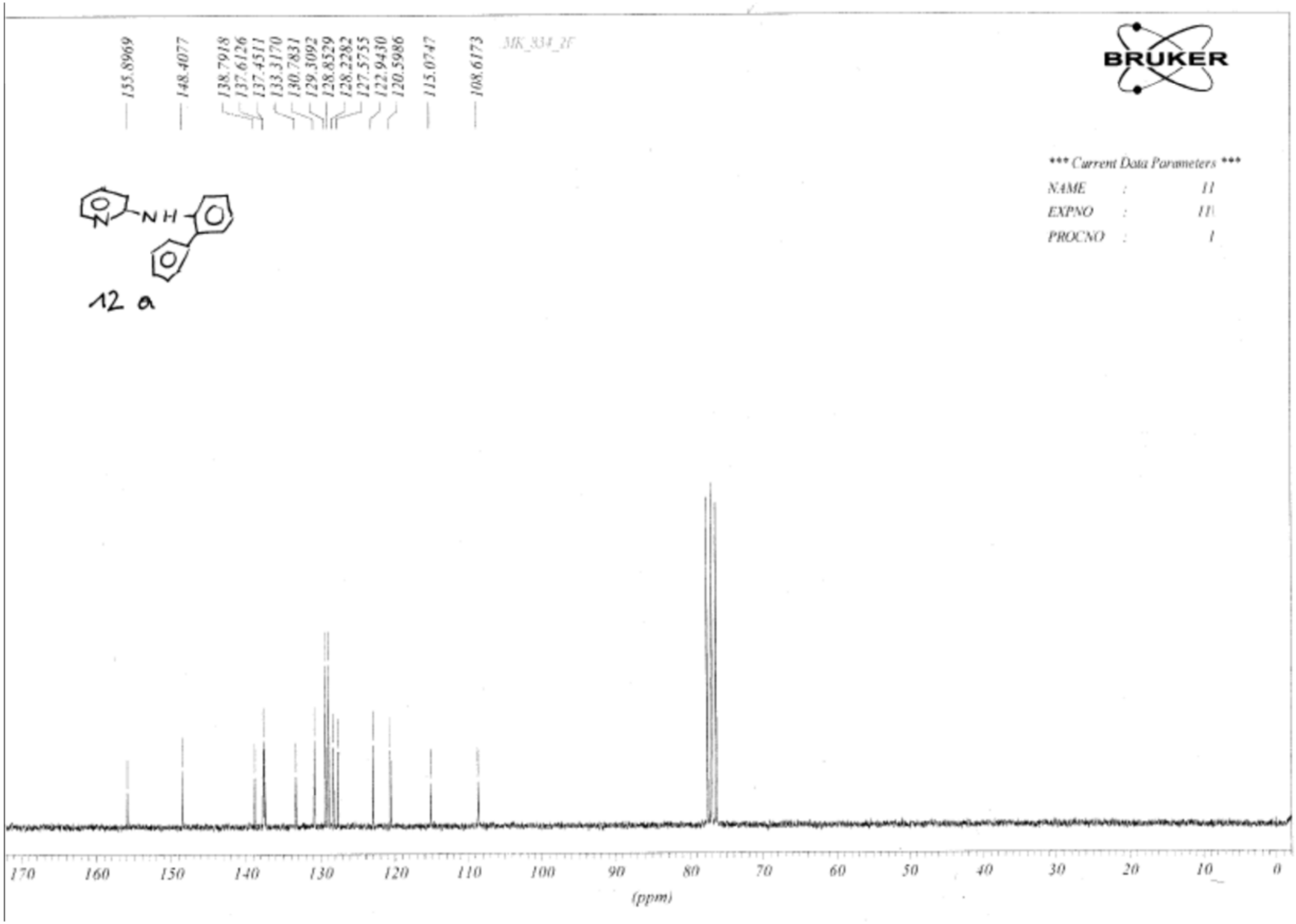
155.8969
148.4077
138.7918
137.6126
137.4511
133.3170
130.7831
129.3092
128.8529
128.2282
127.5755
122.9430
120.5986
115.0747
108.6173
MK_334_2F
BRUKER
*** Current Data Parameters ***
NAME : 11
EXPNO : 11
PROCNO : 1
NH
12 a
170 160 150 140 130 120 110 100 90 80 70 60 50 40 30 20 10 0
(ppm)

***N*-(4'-methyl-[1,1'-biphenyl]-2-yl)pyridin-2-amine (12b):**

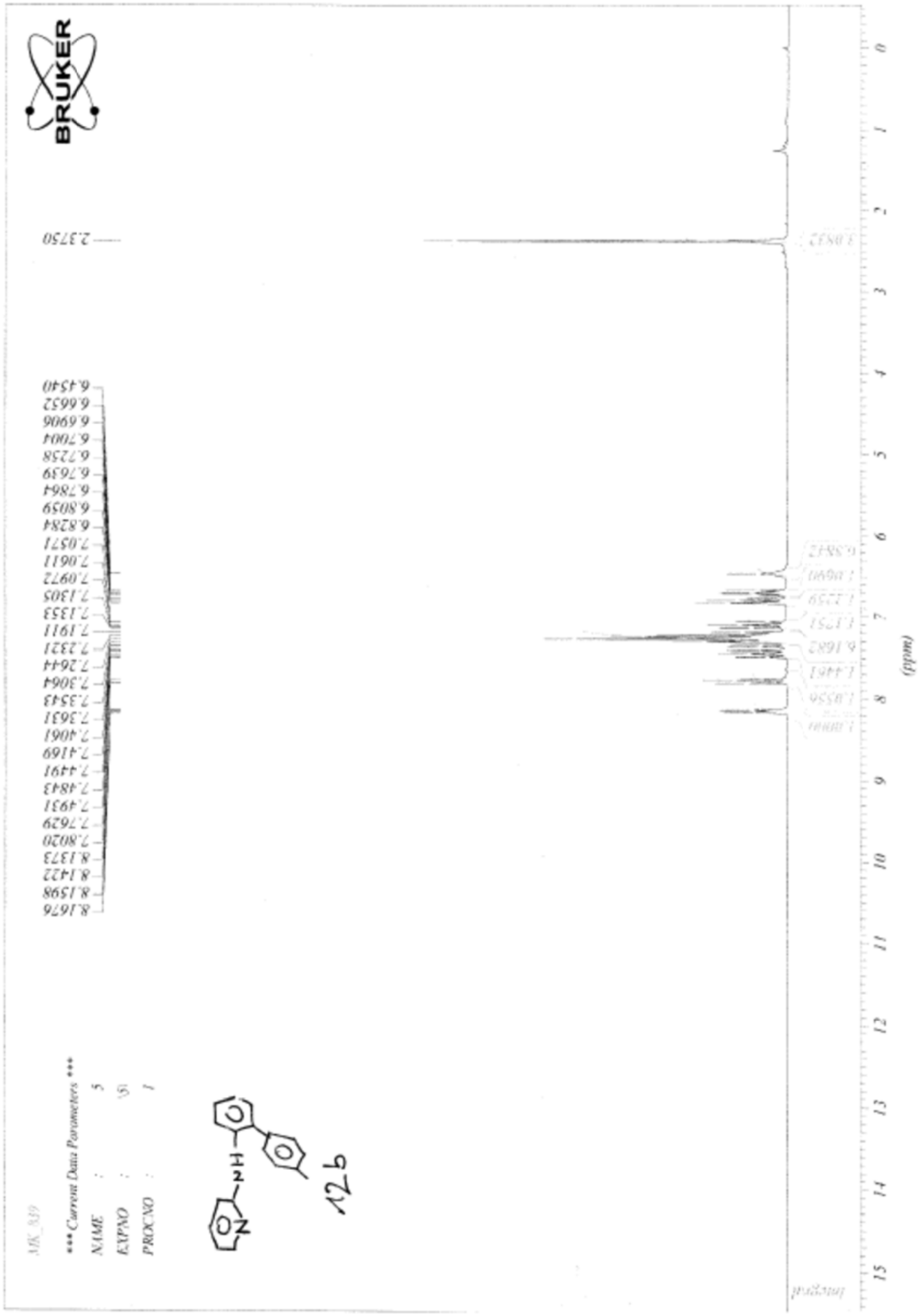

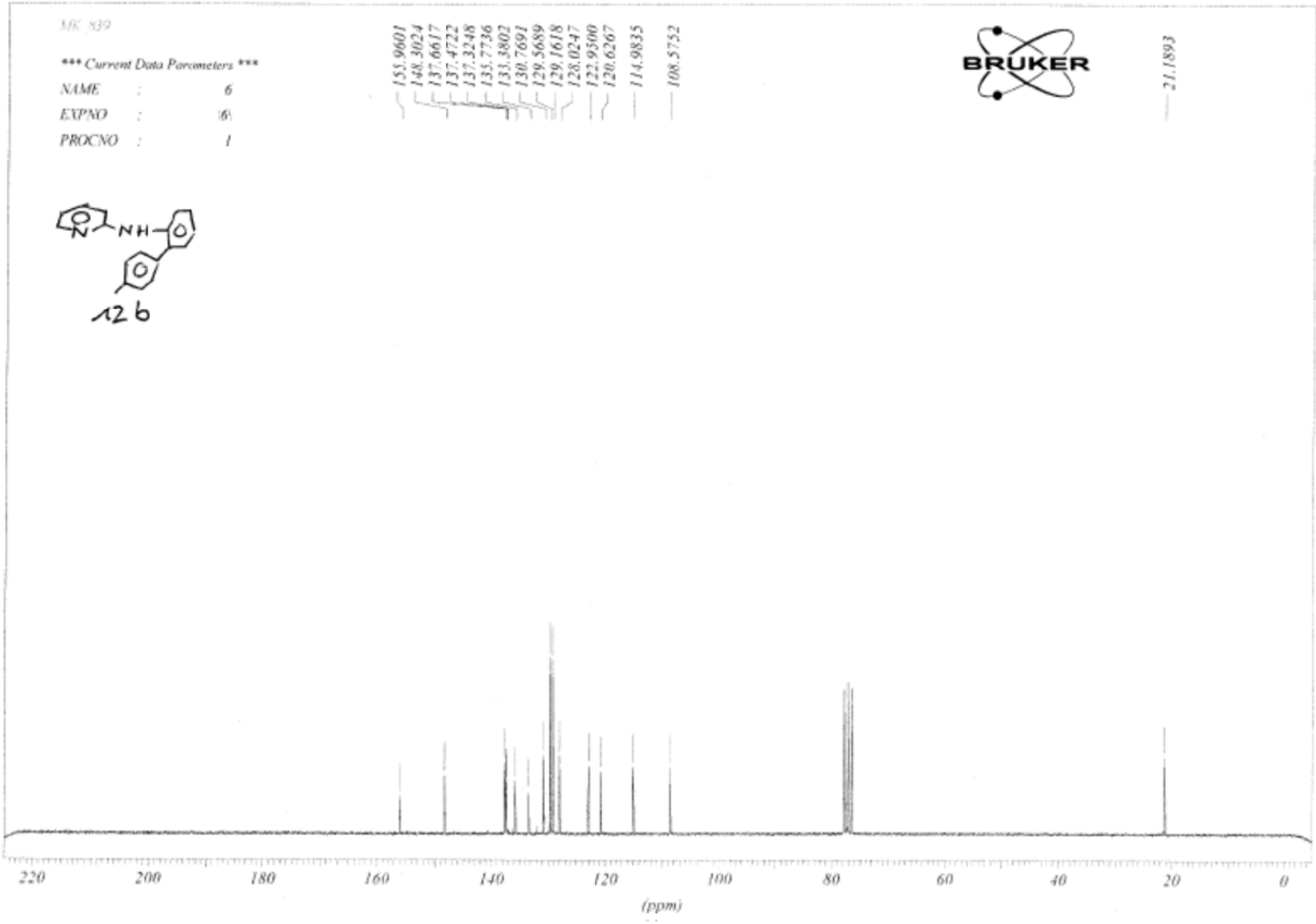
MK 839
*** Current Data Parameters ***
NAME : 6
EXPNO : 16\
PROCNO : 1
NH
12b
155.9601
148.3024
137.6617
137.4722
137.3248
135.7736
133.3802
130.7691
129.5689
129.1618
128.0247
122.9500
120.6267
114.9835
108.5752
21.1893
BRUKER
220 200 180 160 140 120 100 80 60 40 20 0
(ppm)

N-(4'-(*tert*-butyl)-[1,1'-biphenyl]-2-yl)pyridin-2-amine (12c):

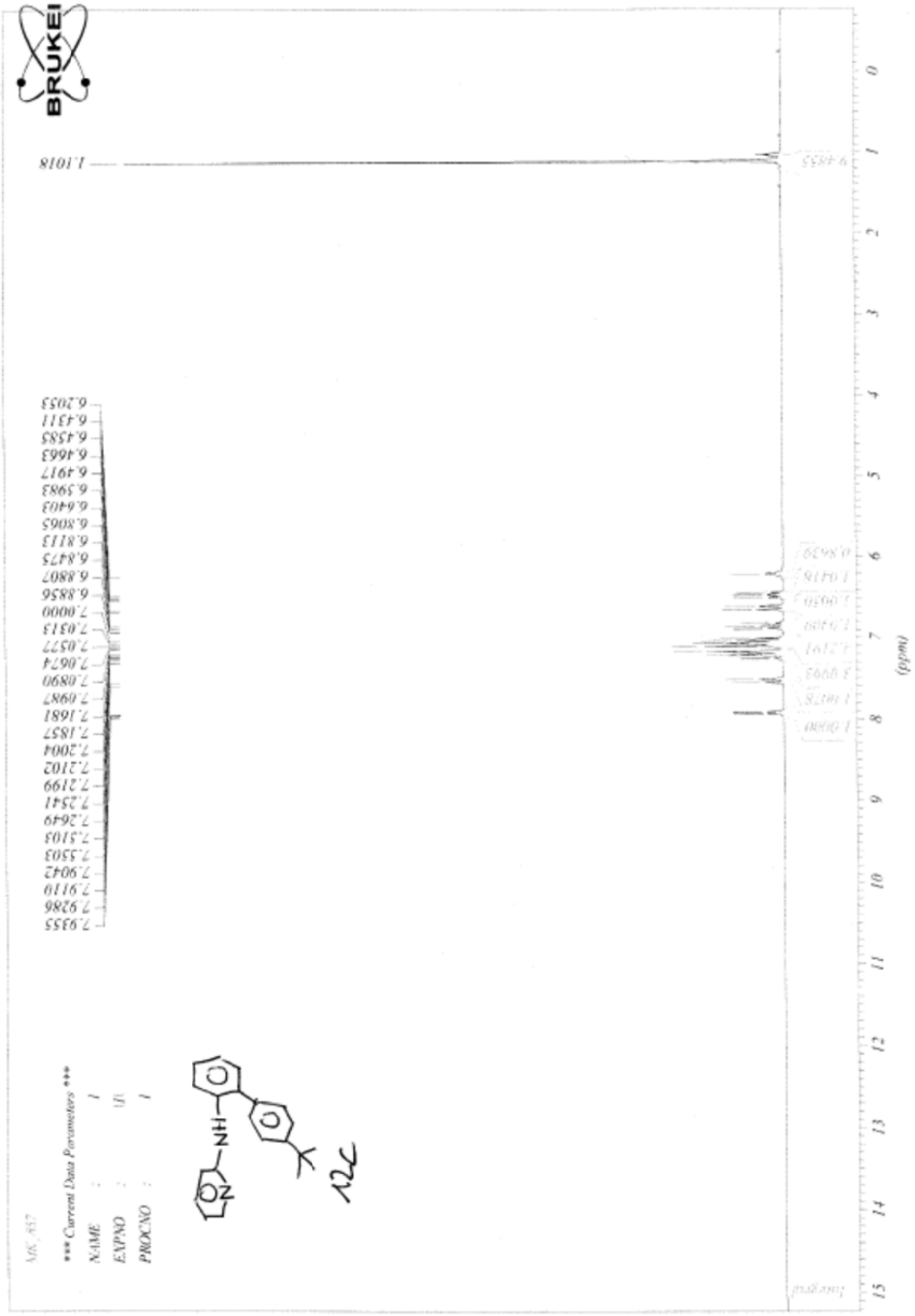

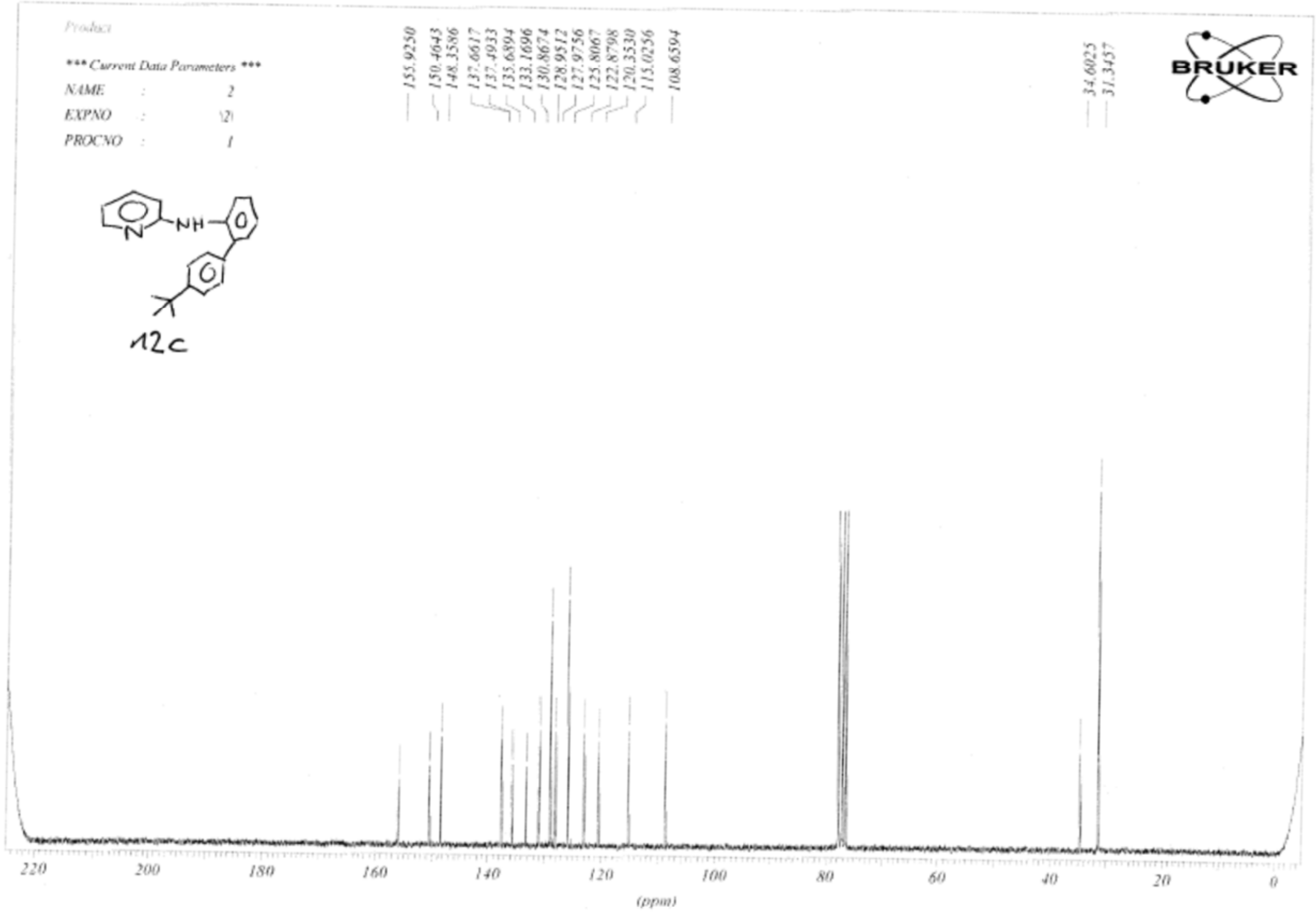
Product
*** Current Data Parameters ***
NAME : 2
EXPNO : \2\
PROCNO : 1
NH
N
12c
155.9250
150.4643
148.3586
137.6617
137.4933
135.6894
133.1696
130.8674
128.9512
127.9756
125.8067
122.8798
120.3530
115.0256
108.6594
34.6025
31.3457
BRUKER
220
200
180
160
140
120
100
80
60
40
20
0
(ppm)

***N*-(4'-chloro-[1,1'-biphenyl]-2-yl)pyridin-2-amine (12d):**

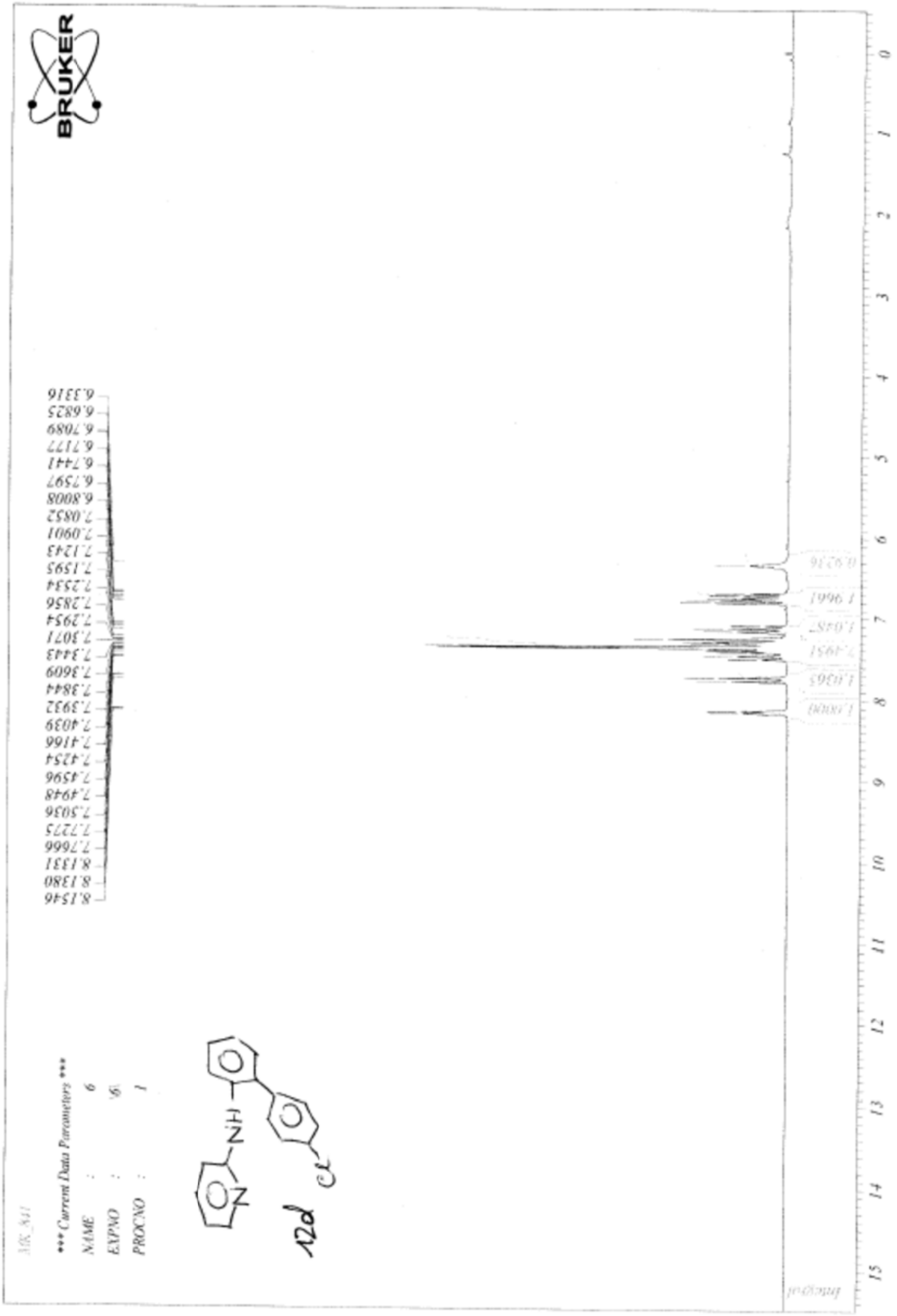

MK_841

*** Current Data Parameters ***

NAME : 7

EXPNO : 171

PROCNO : 1

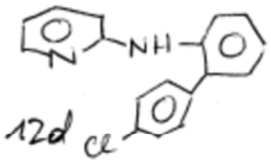

12d

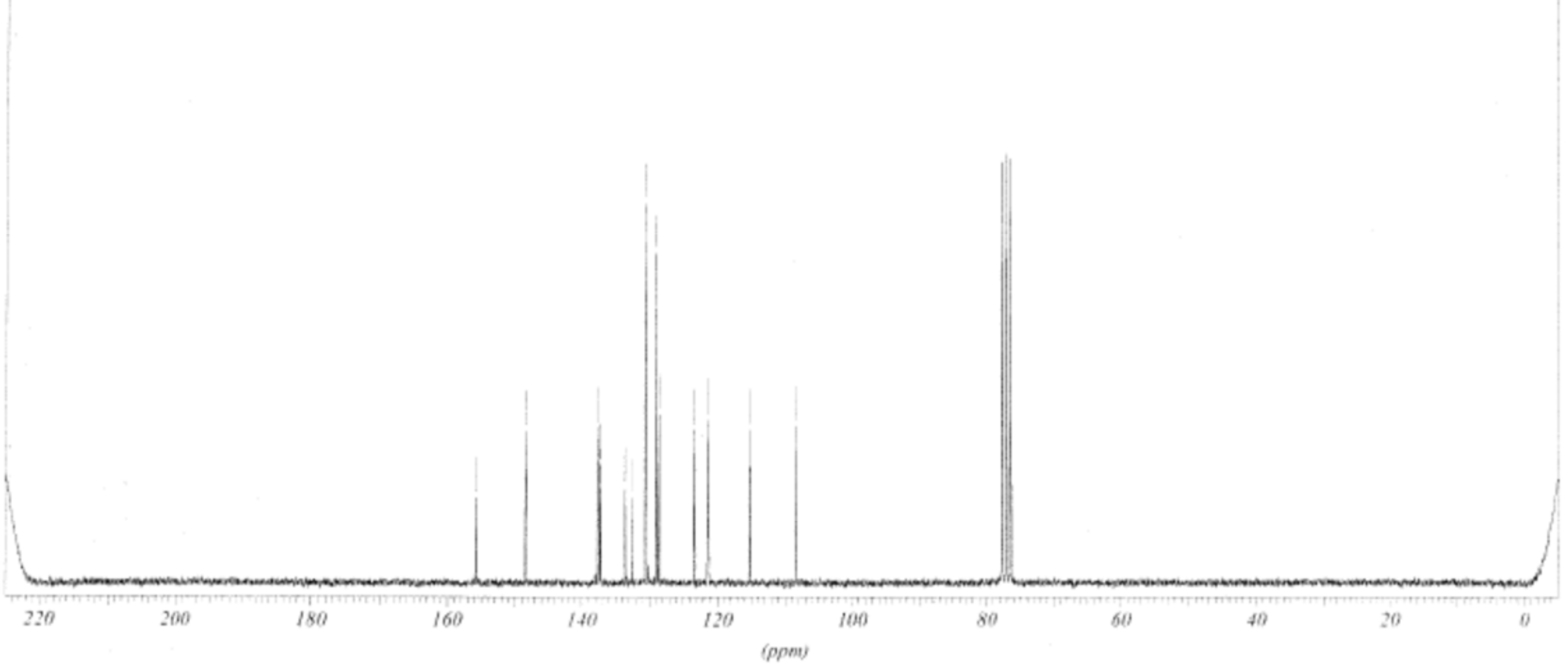

***N*-(3'-nitro-[1,1'-biphenyl]-2-yl)pyridin-2-amine (12e):**

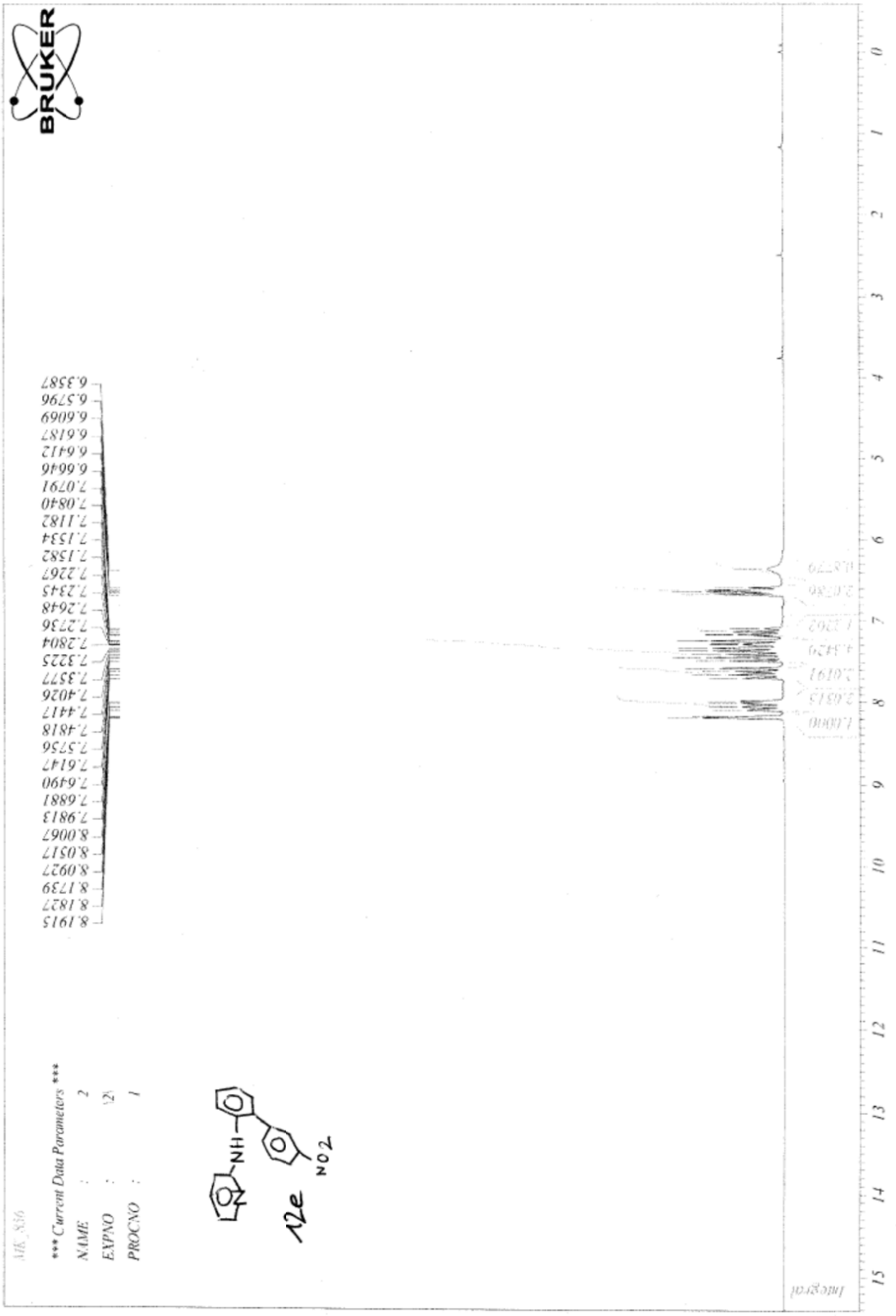

MK 856

*** Current Data Parameters ***

NAME	:	3
EXPNO	:	3
PROCNO	:	1

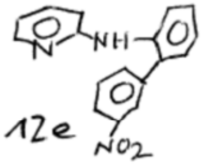

12e

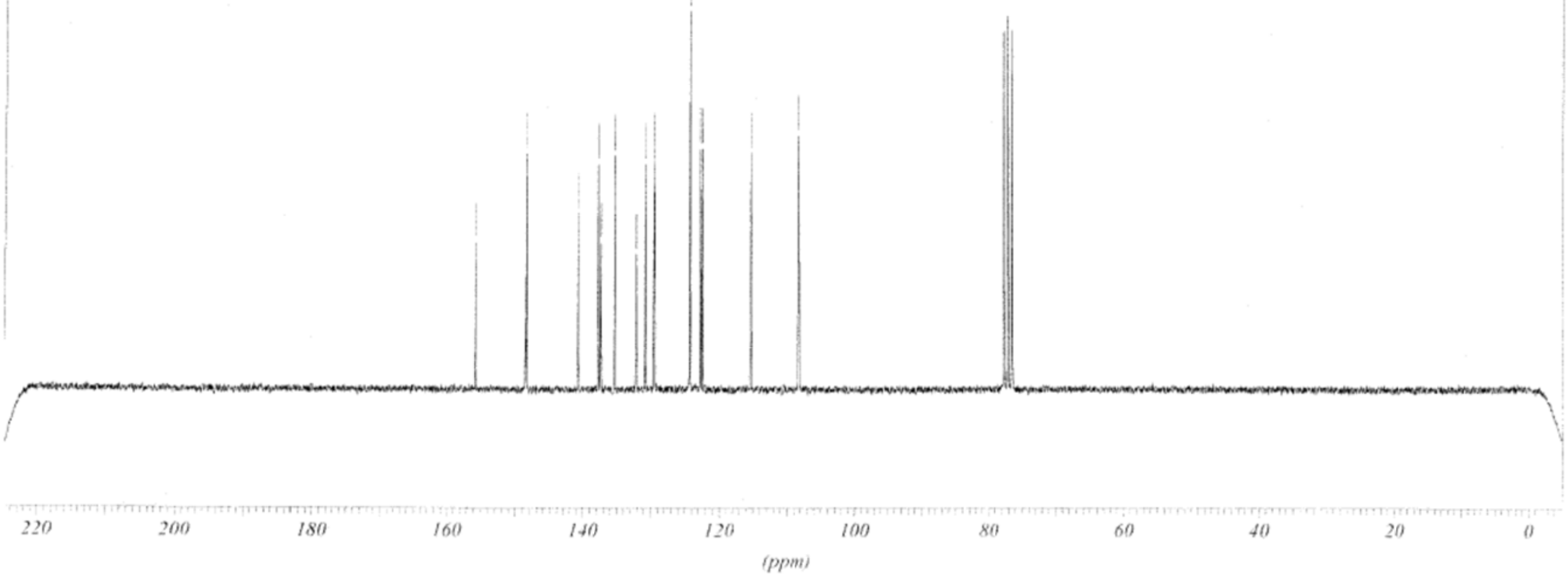

***N*-(5-methoxy-[1,1'-biphenyl]-2-yl)pyridin-2-amine (12f):**

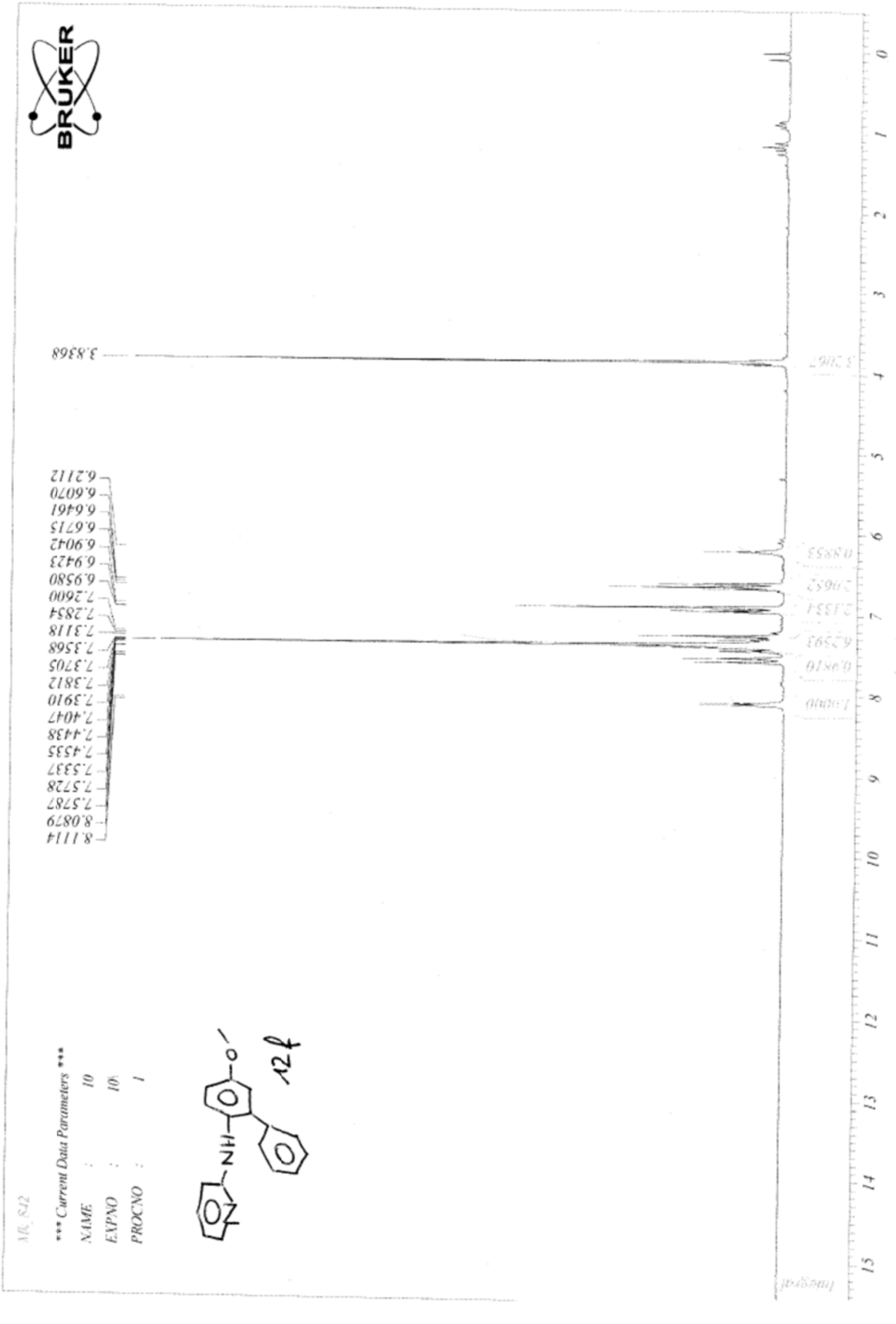

Mk_842

*** Current Data Parameters ***

NAME : 11

EXPNO : 11\

PROCNO : 1

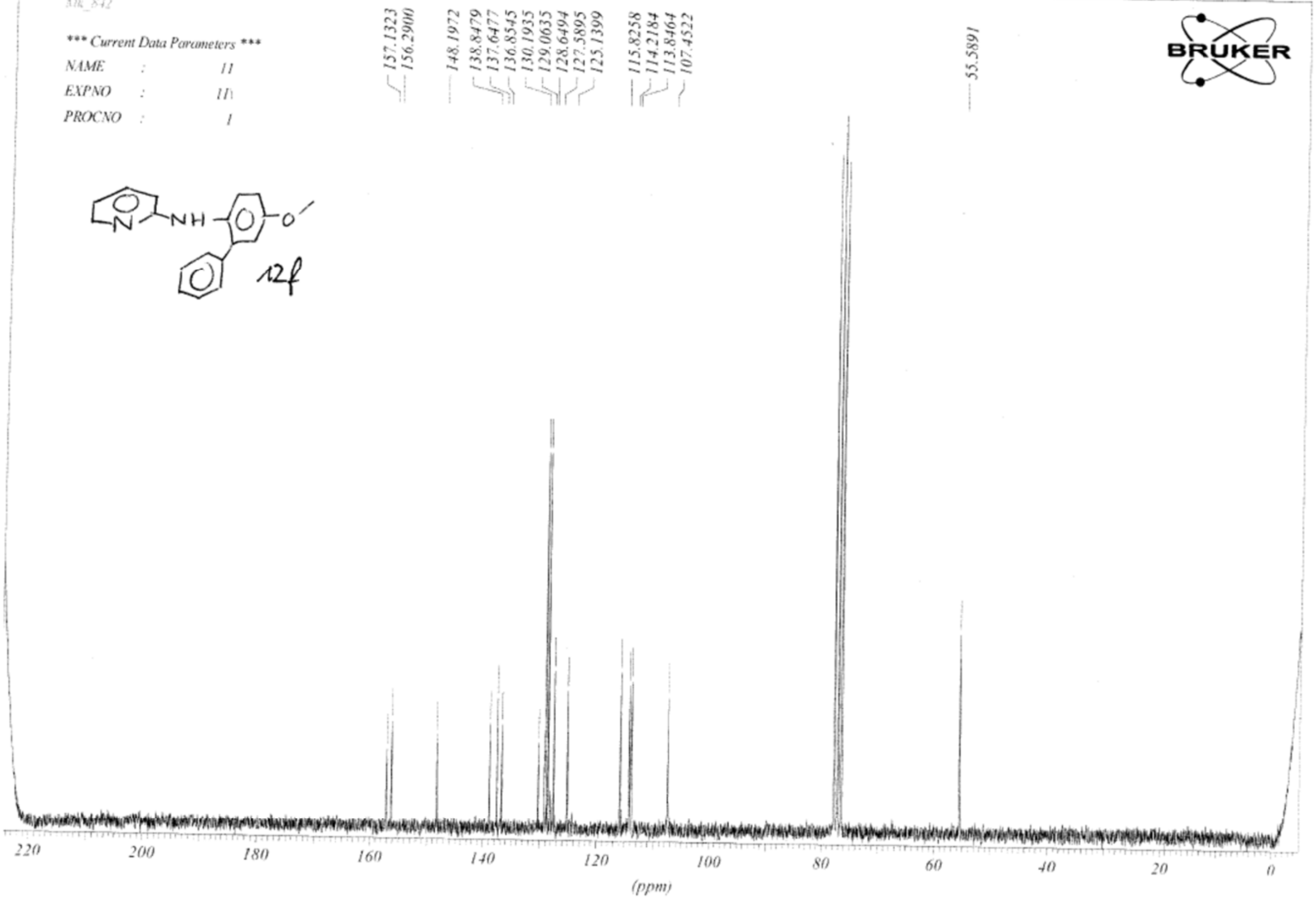

***N*-(5-methoxy-4'-methyl-[1,1'-biphenyl]-2-yl)pyridin-2-amine (12g):**

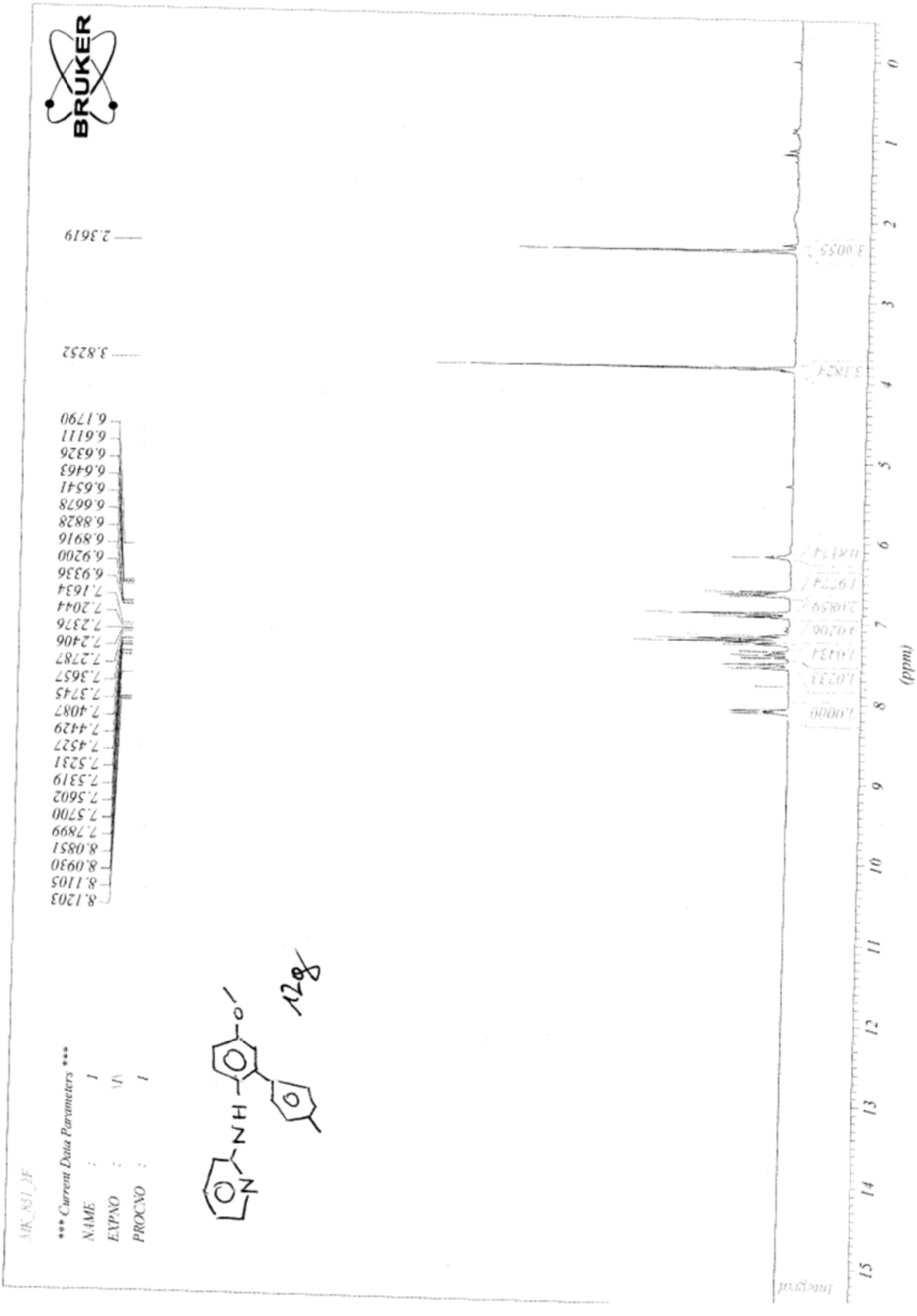

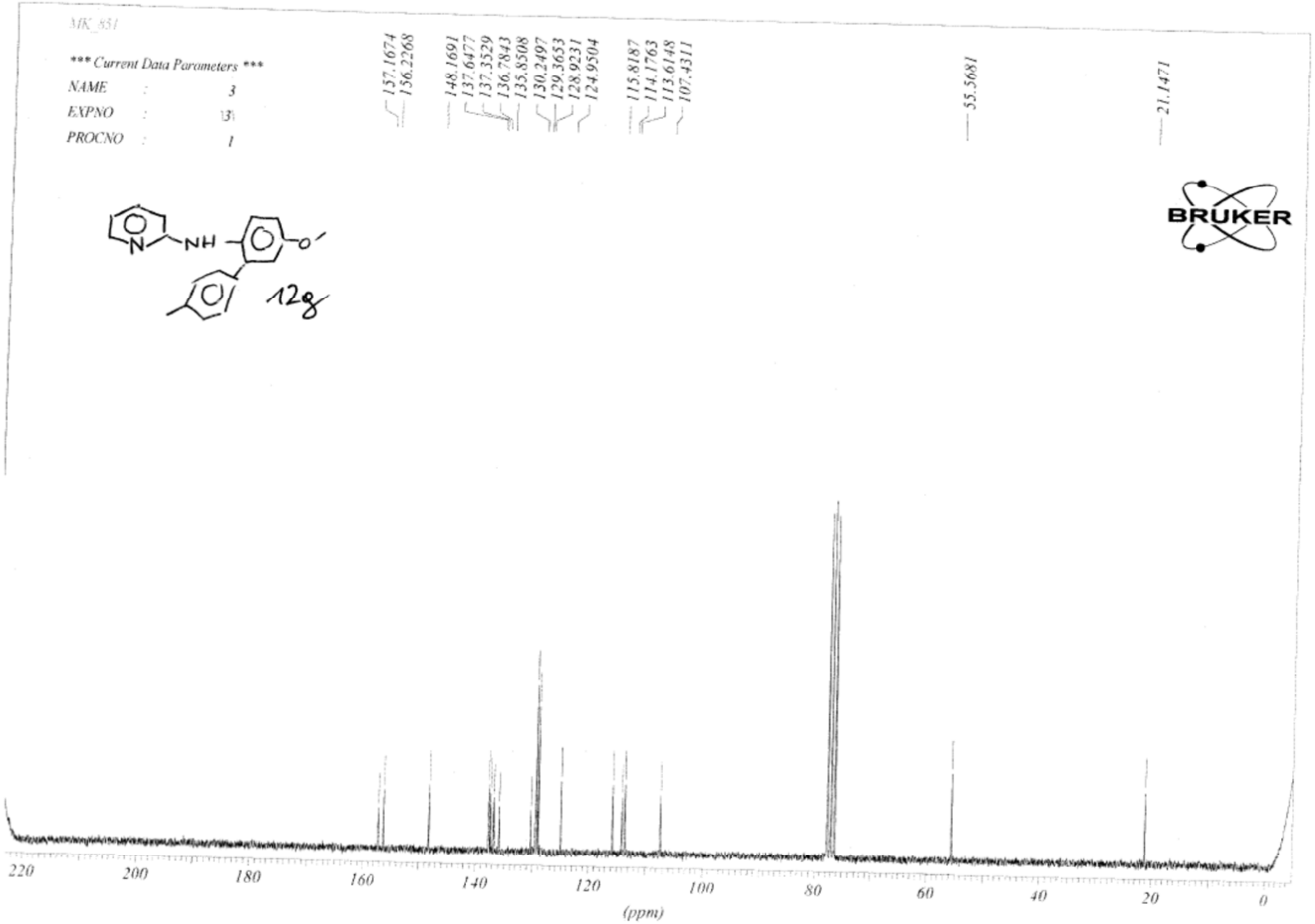
MK_851
*** Current Data Parameters ***
NAME : 3
EXPNO : 3\
PROCNO : 1
NH
12g
157.1674
156.2268
148.1691
137.6477
137.3529
136.7843
135.8508
130.2497
129.3653
128.9231
124.9504
115.8187
114.1763
113.6148
107.4311
55.5681
21.1471
220
200
180
160
140
120
100
80
60
40
20
0
(ppm)

***N*-(5-methoxy-3'-nitro-[1,1'-biphenyl]-2-yl)pyridin-2-amine (12h):**

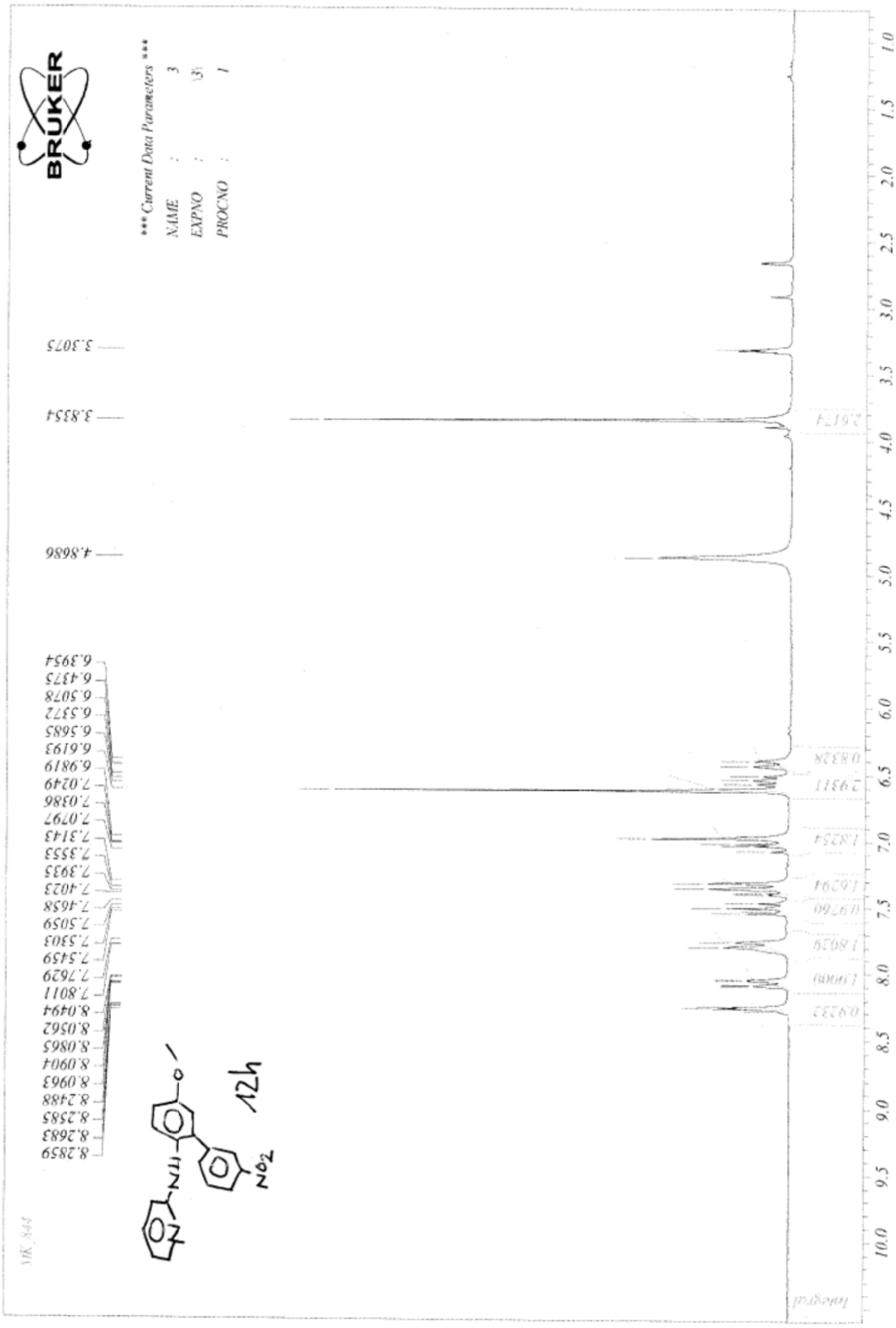

MK_844

*** Current Data Parameters ***

NAME : KO5B0B~1

EXPNO : 4

PROCNO : 1

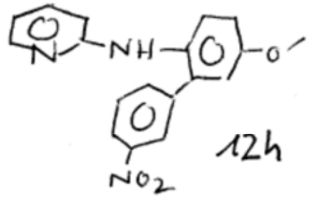

12h

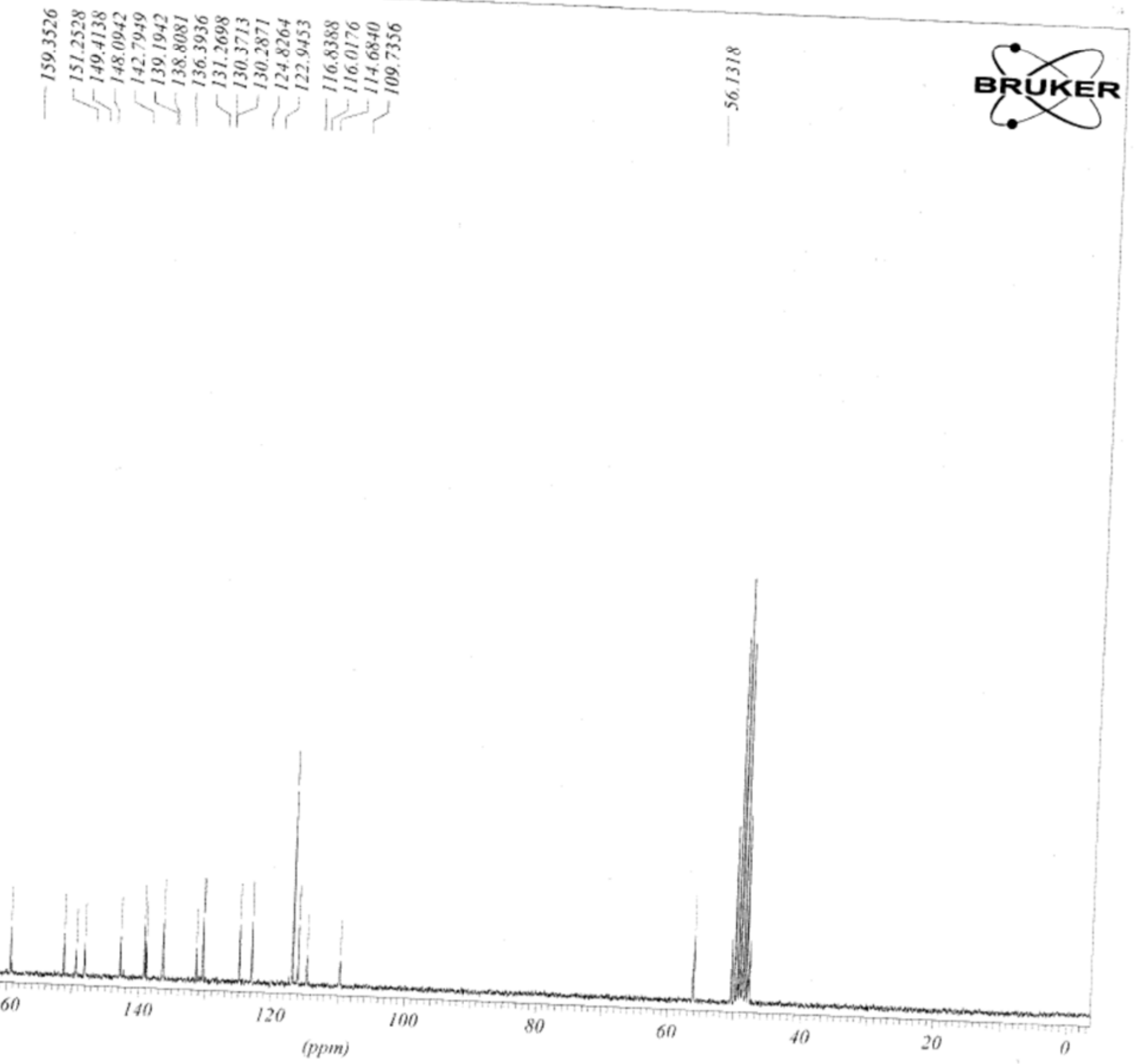

MK_844

*** Current Data Parameters ***

NAME : KO5B0B~1

EXPNO : 4

PROCNO : 1

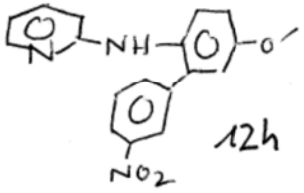

12h

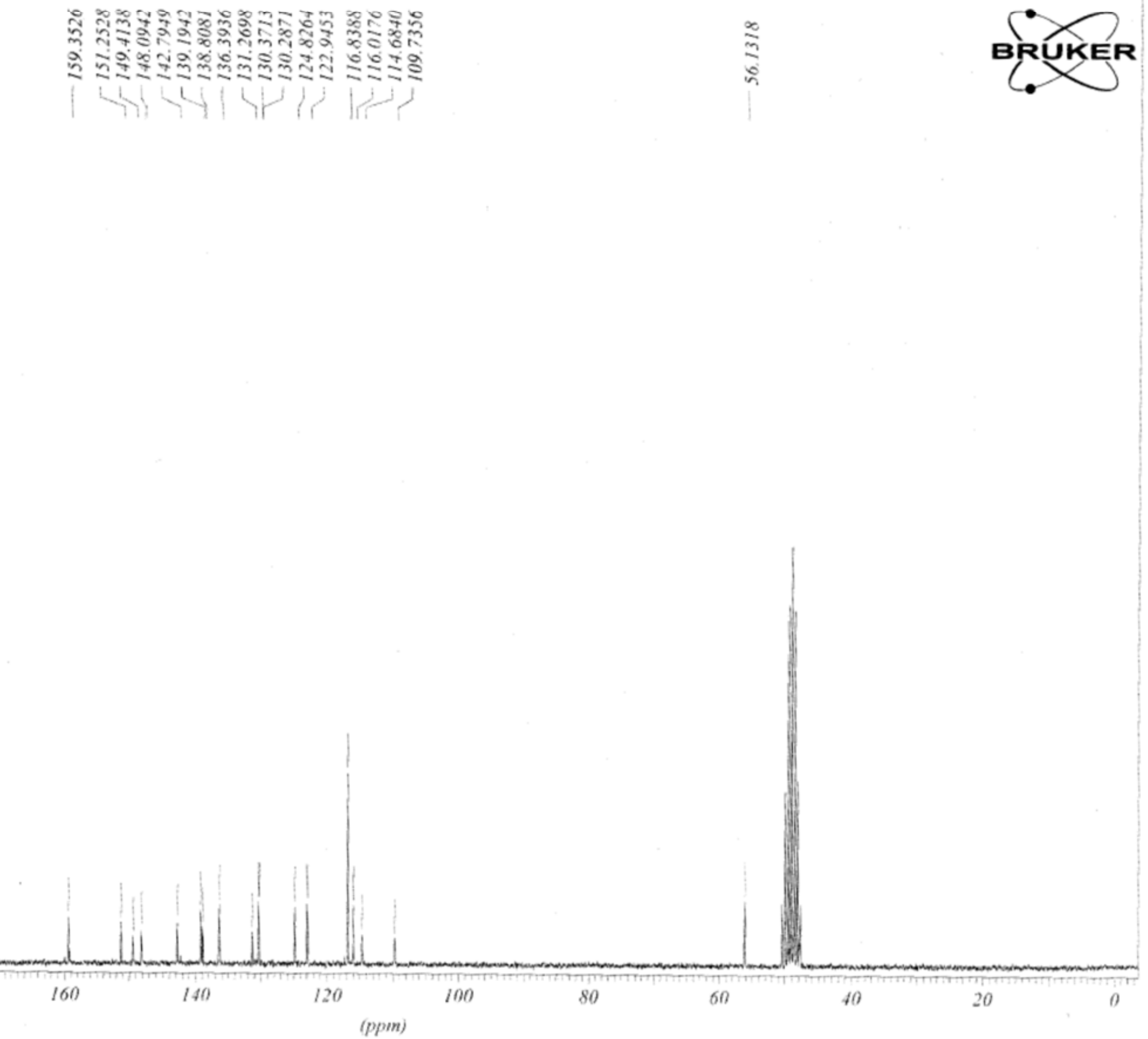

5'-Methoxy-2'-(pyridin-2-ylamino)-[1,1'-biphenyl]-4-yl acetate (12i):

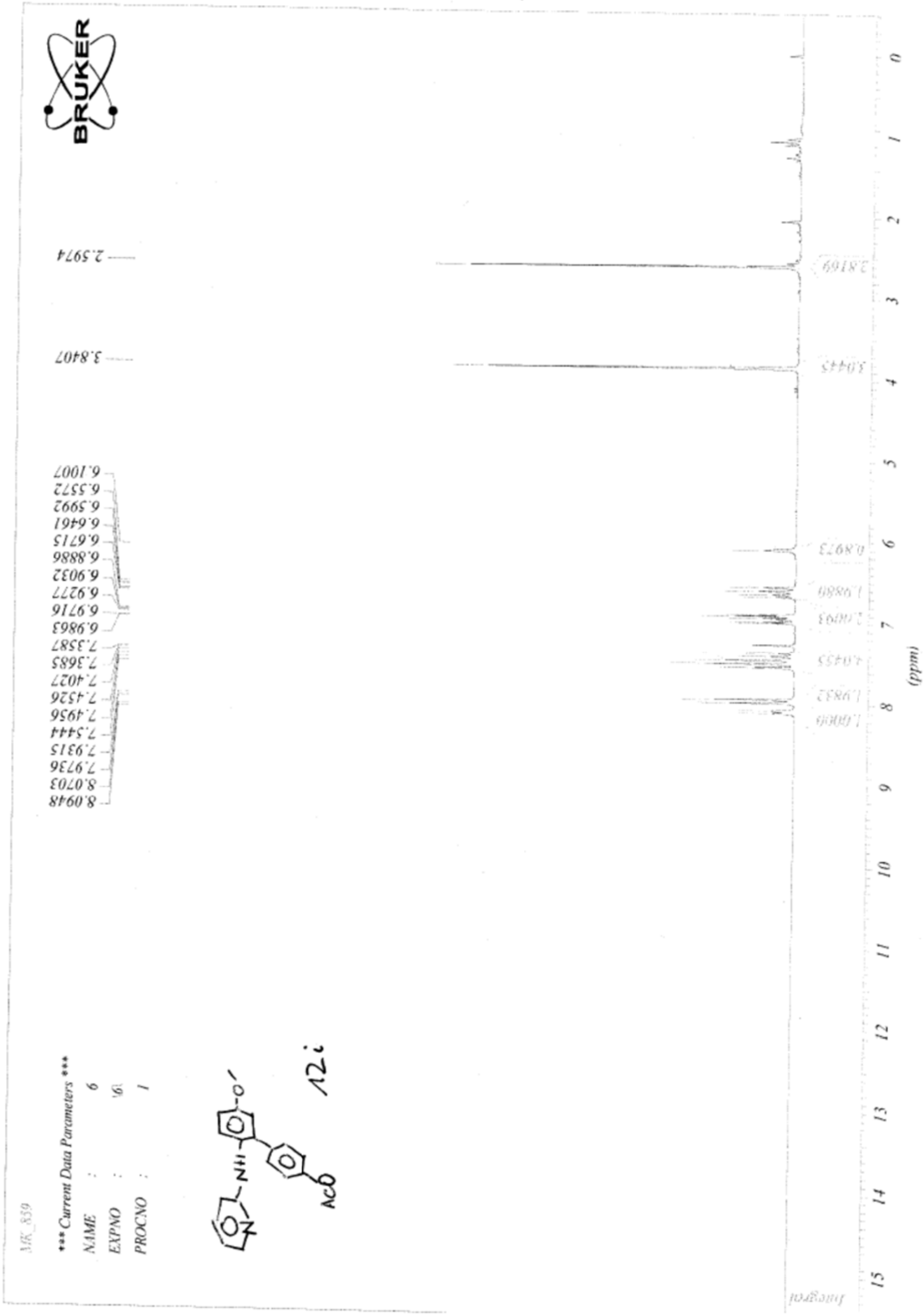

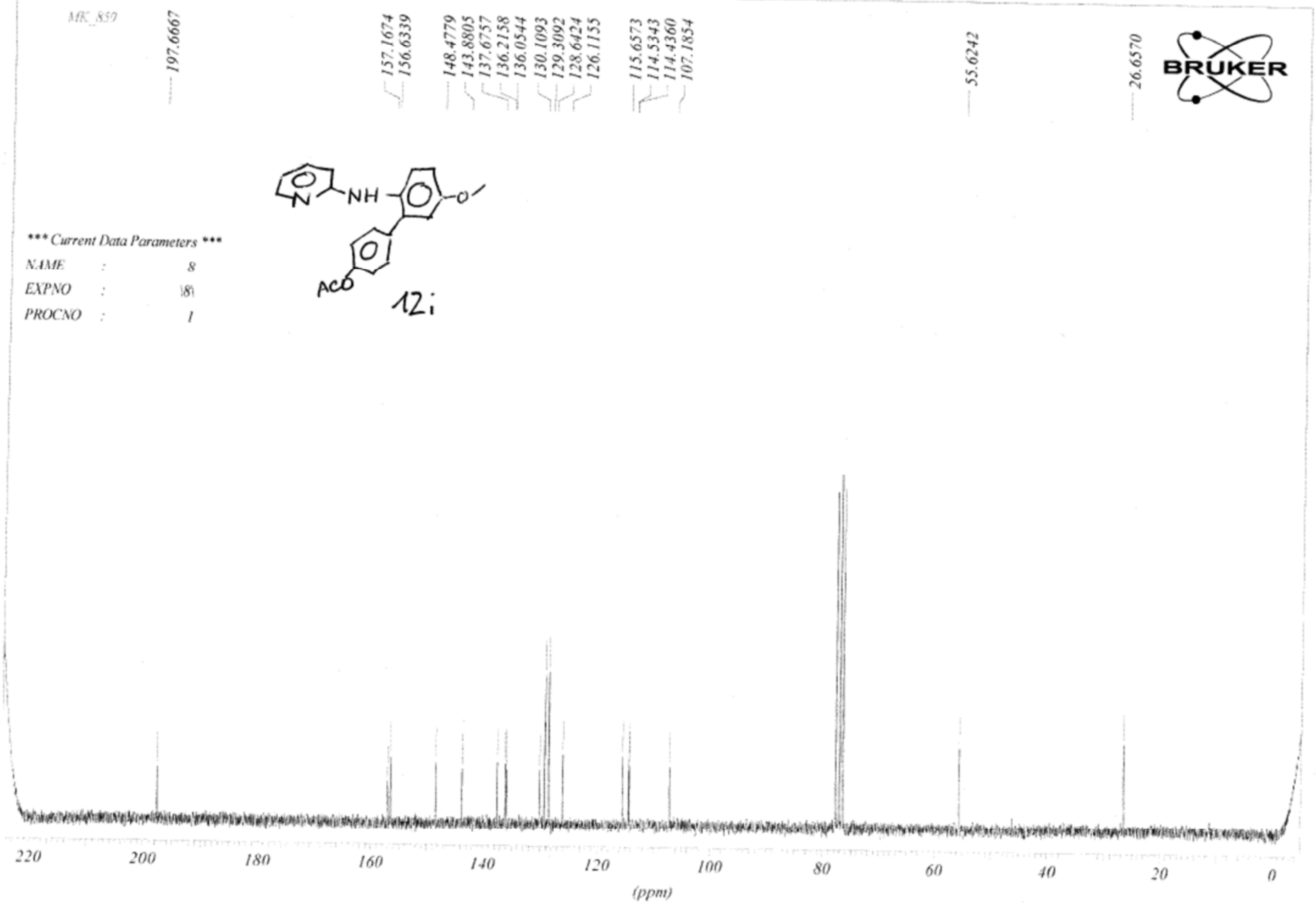
MK_850
197.6667
157.1674
156.6339
148.4779
143.8805
137.6757
136.2158
136.0544
130.1093
129.3092
128.6424
126.1155
115.6573
114.5343
114.4360
107.1854
55.6242
26.6570
*** Current Data Parameters ***
NAME : 8
EXPNO : 18
PROCNO : 1
NH
AcO
12i
220
200
180
160
140
120
100
80
60
40
20
0
(ppm)

***N*-(4'-fluoro-5-methoxy-[1,1'-biphenyl]-2-yl)pyridin-2-amine (12j):**

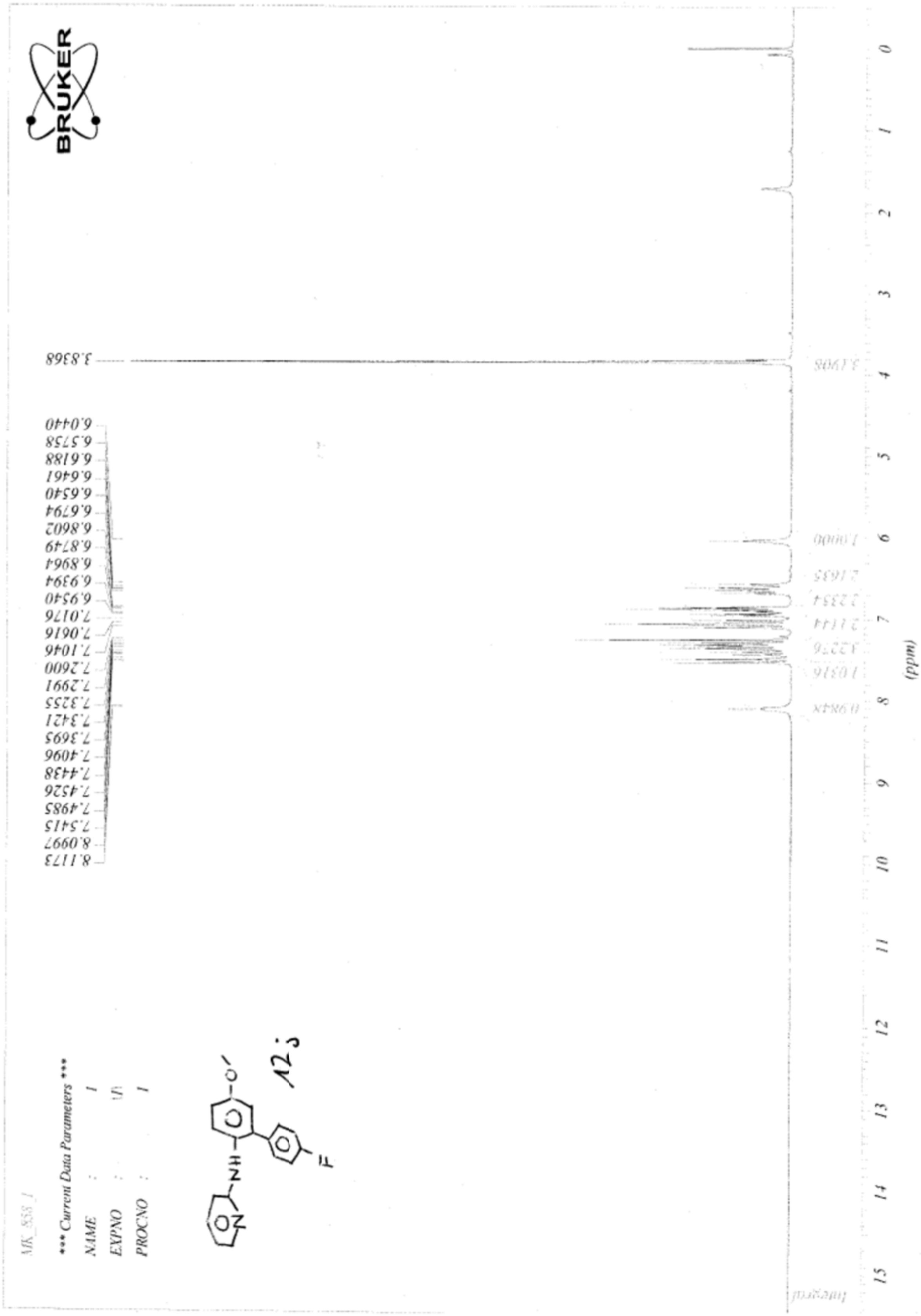

MK_858_1

*** Current Data Parameters ***

NAME : 4

EXPNO : 14

PROCNO : 1

12j

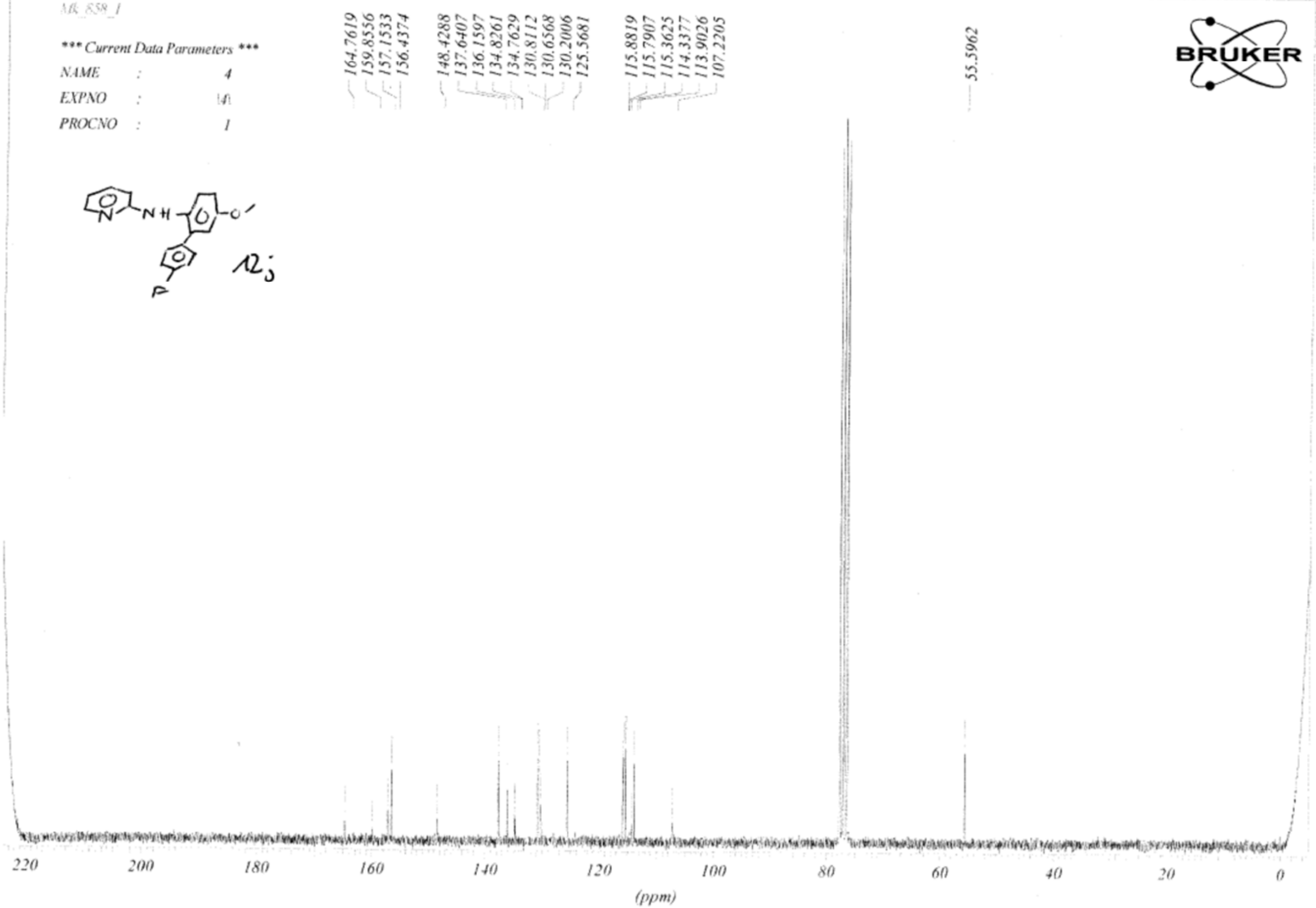

***N*-(5-chloro-[1,1'-biphenyl]-2-yl)pyridin-2-amine (12k):**

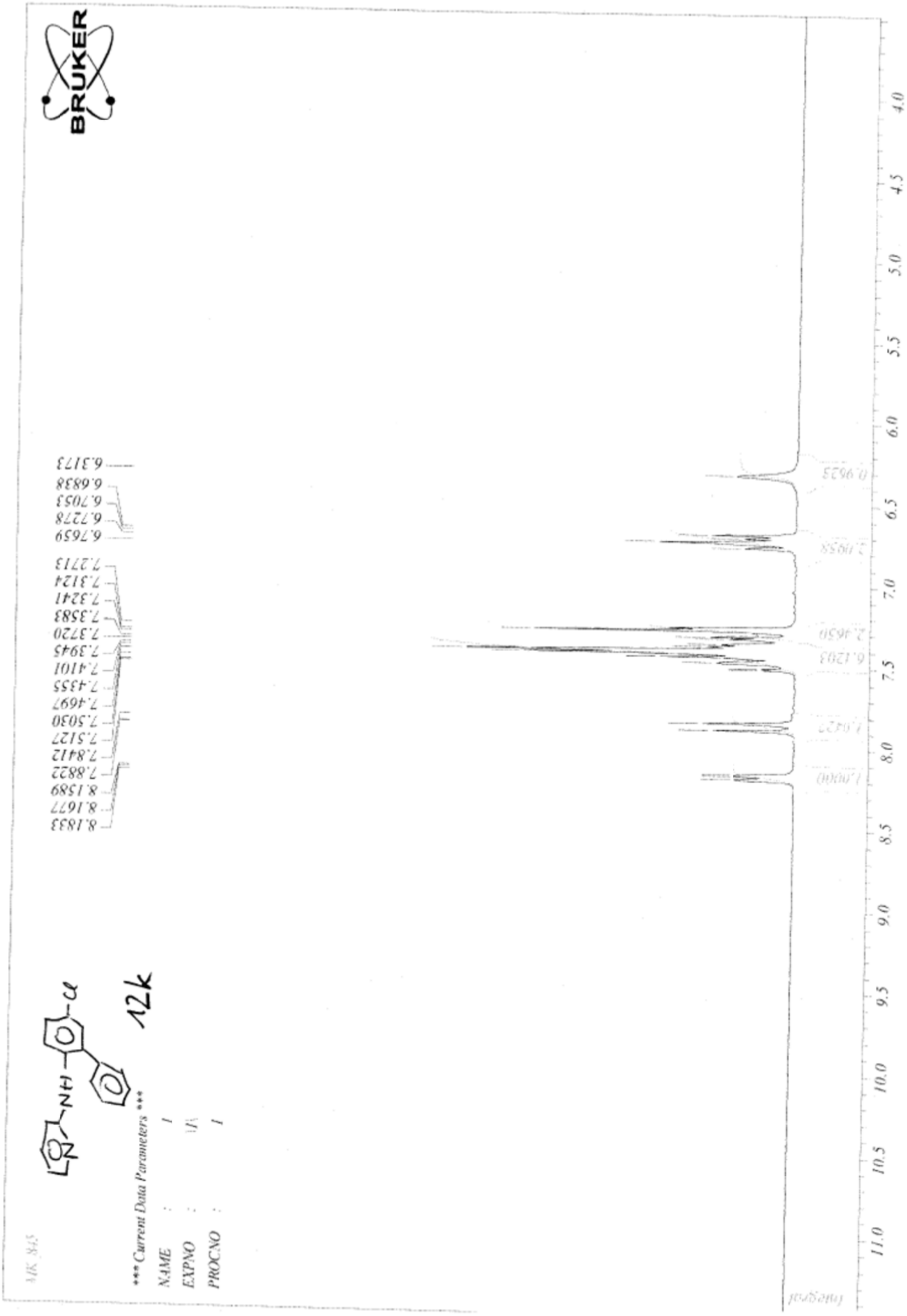

MK_845

*** Current Data Parameters ***

NAME : 2

EXPNO : 21

PROCNO : 1

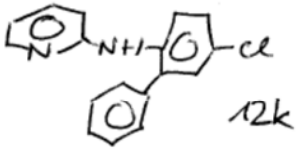

12k

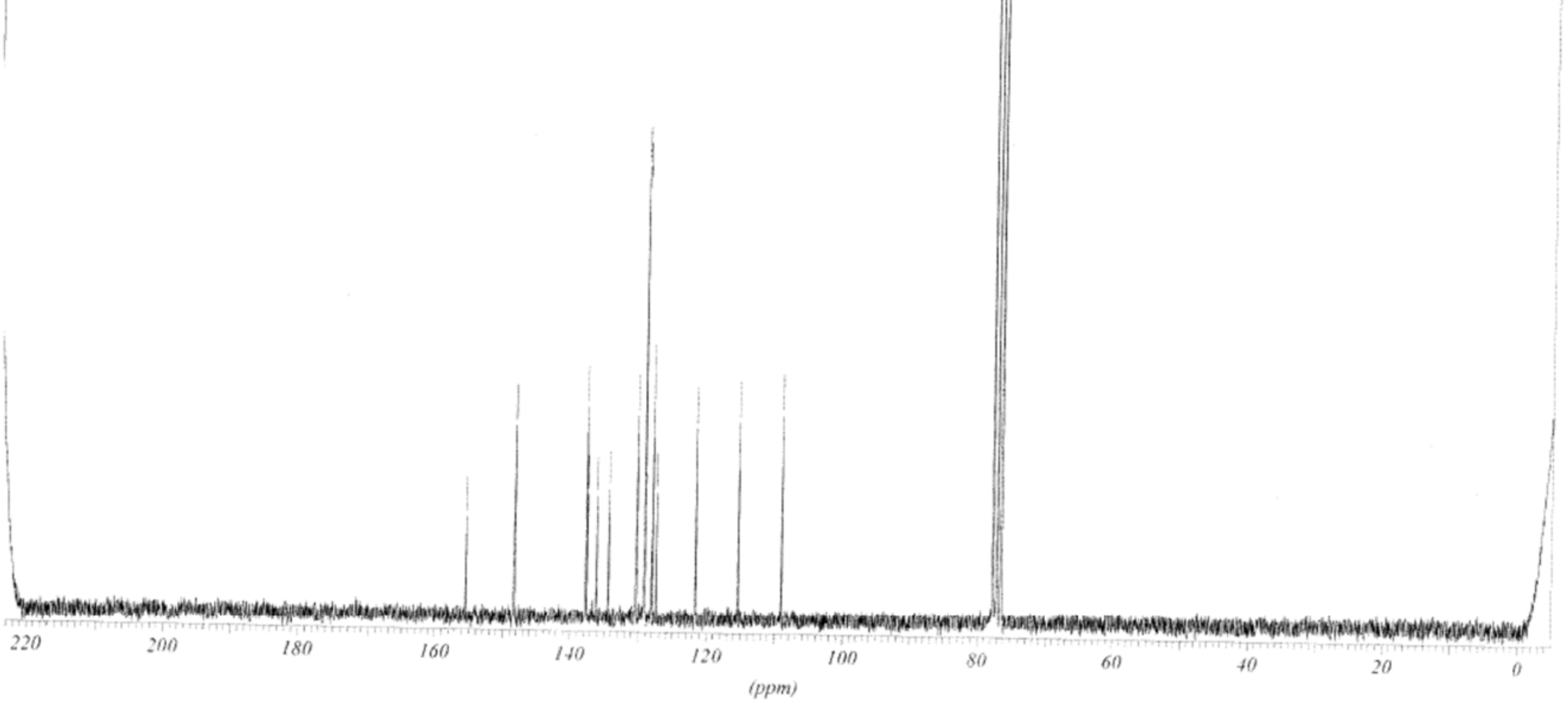

***N*-(5-chloro-4'-methyl-[1,1'-biphenyl]-2-yl)pyridin-2-amine (12l):**

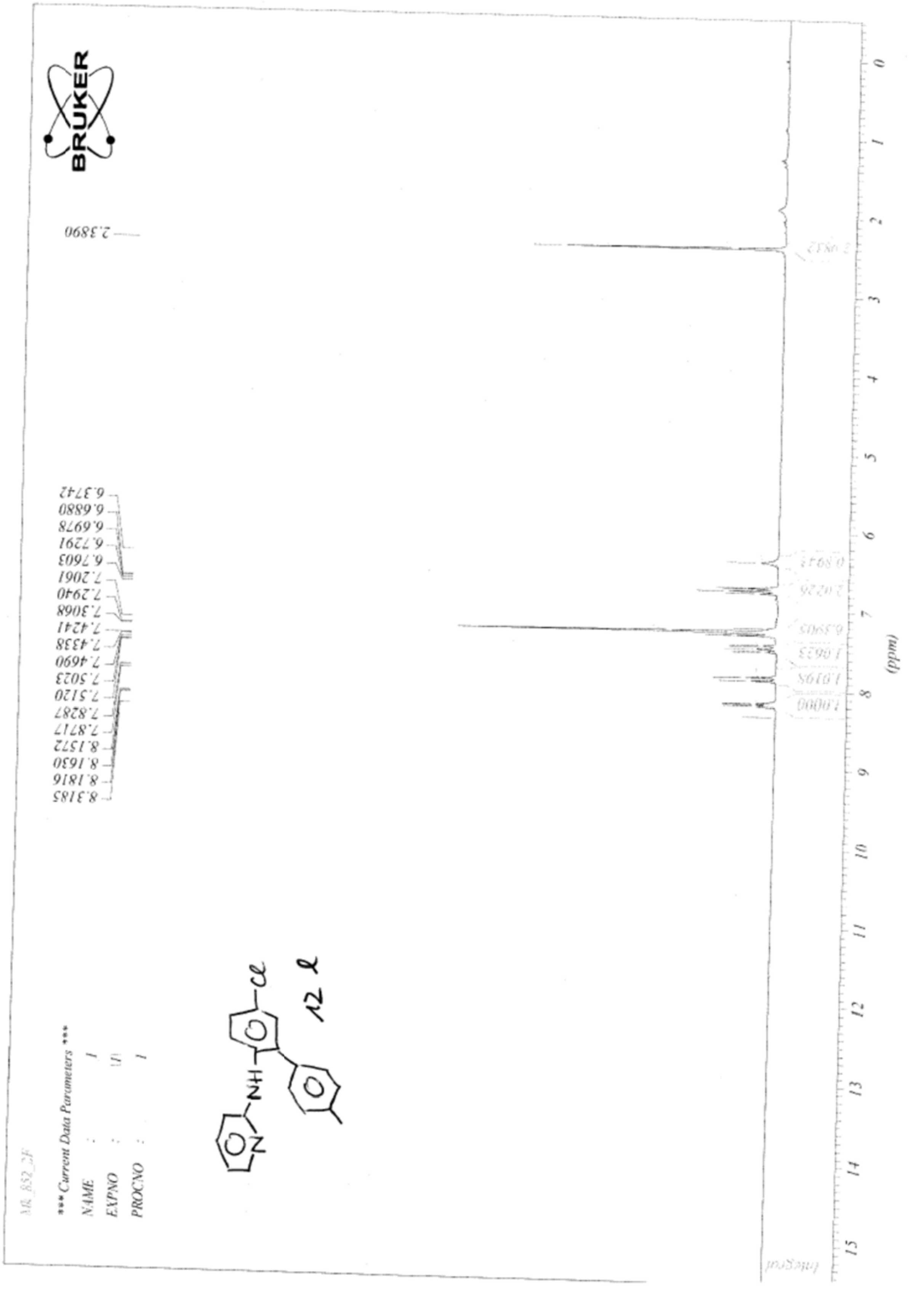

MK_851

*** Current Data Parameters ***

NAME : KO5BE2~1

EXPNO : 2

PROCNO : 1

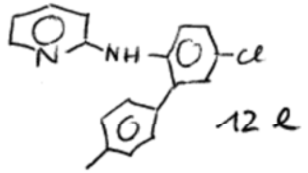

12 l

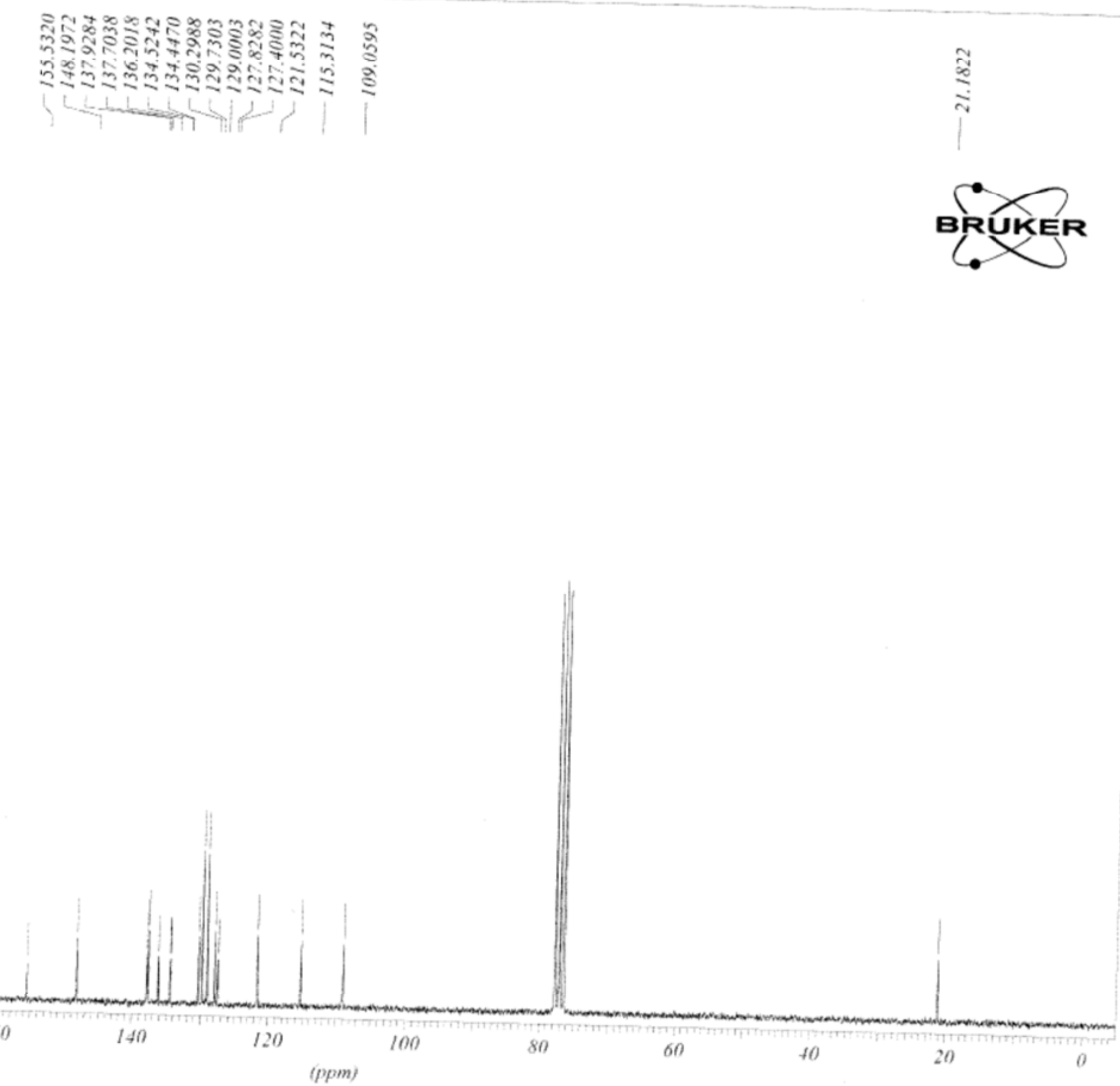

***N*-(4-chloro-[1,1'-biphenyl]-2-yl)pyridin-2-amine (12m):**

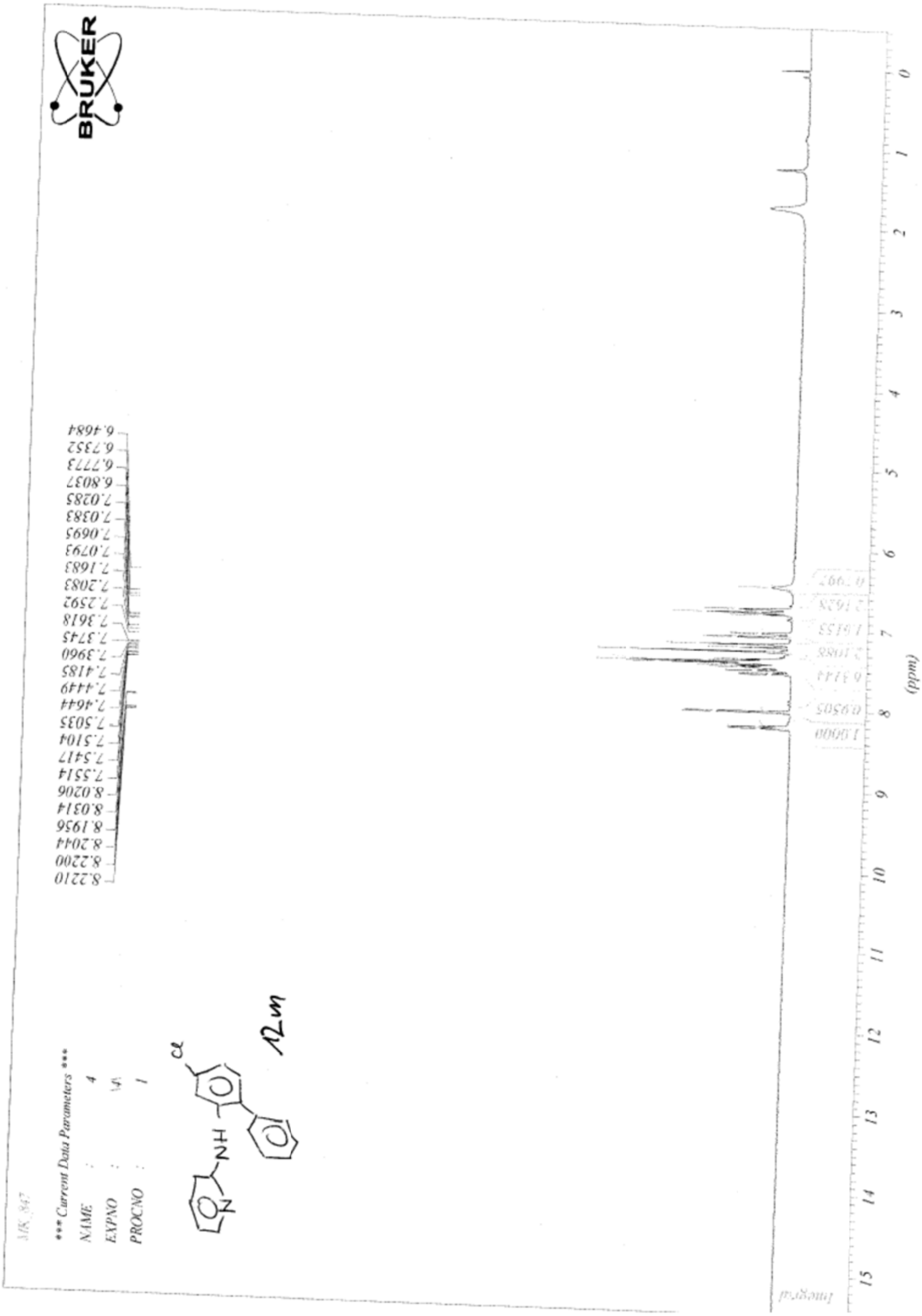

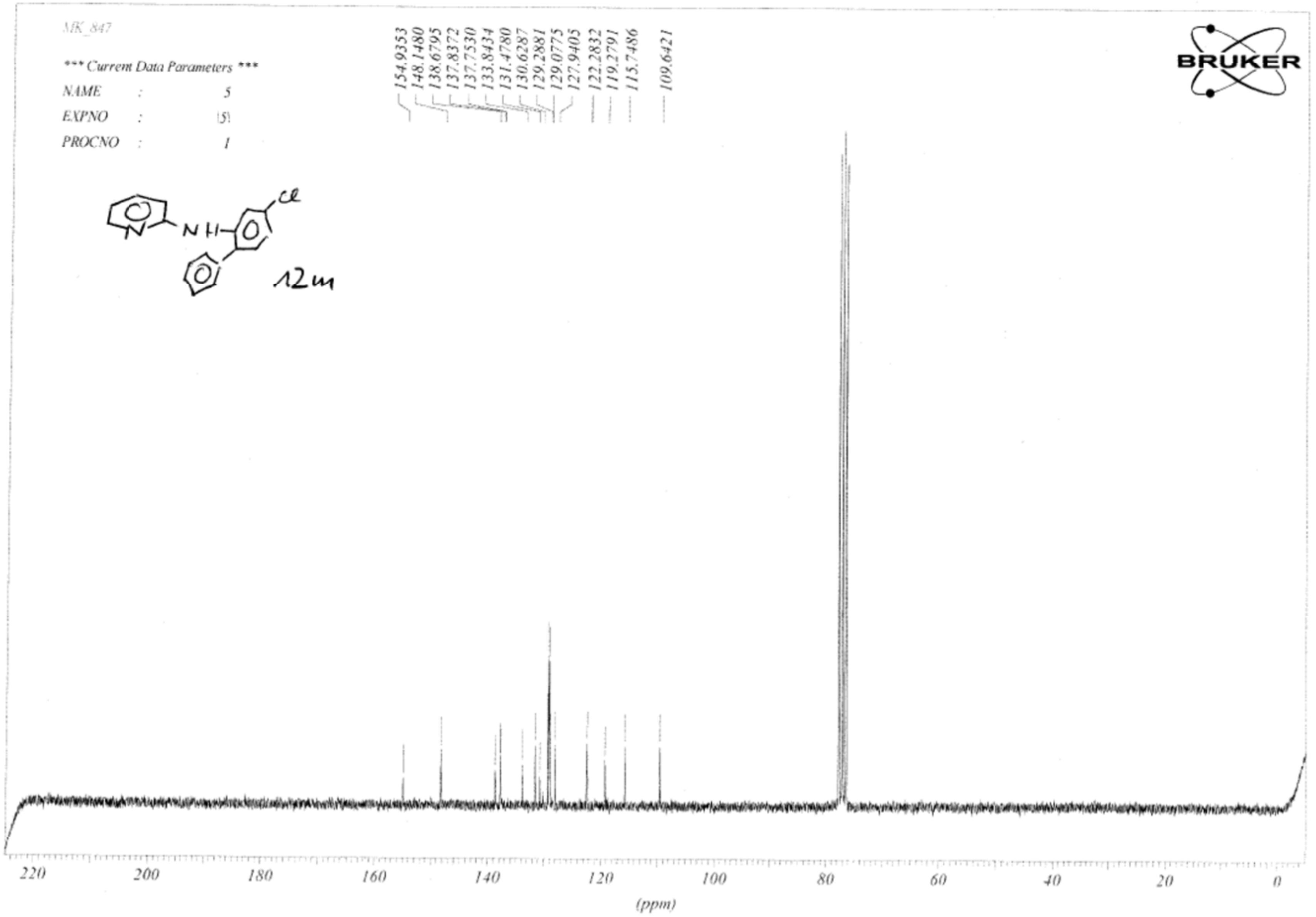
MK_847
*** Current Data Parameters ***
NAME : 5
EXPNO : 15
PROCNO : 1
154.9353
148.1480
138.6795
137.8372
137.7530
133.8434
131.4780
130.6287
129.2881
129.0775
127.9405
122.2832
119.2791
115.7486
109.6421
BRUKER
Cl
NH
12m
220
200
180
160
140
120
100
80
60
40
20
0
(ppm)

Ethyl 6-(pyridin-2-ylamino)-[1,1'-biphenyl]-3-carboxylate (12n):

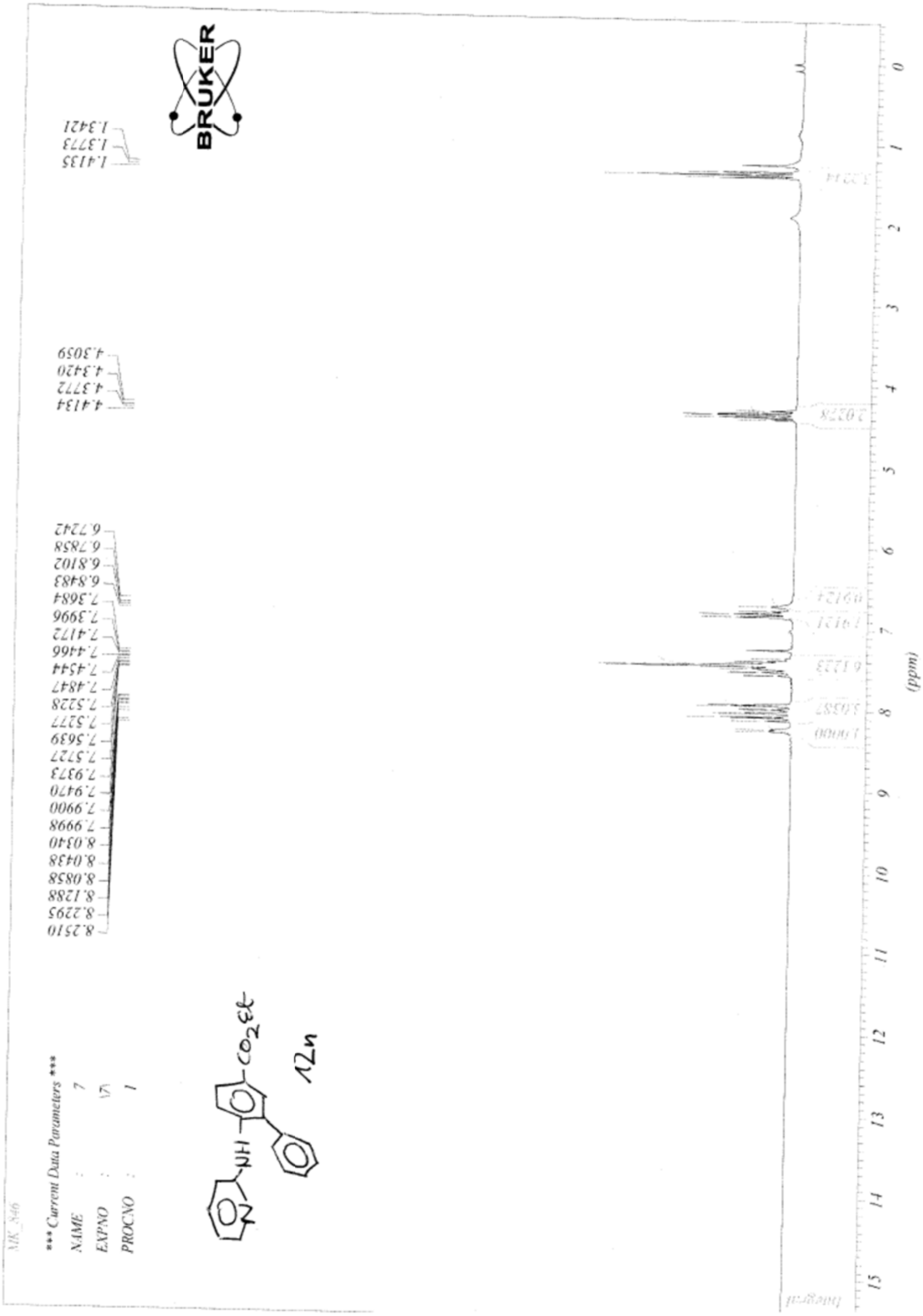

*** Current Data Parameters ***

NAME : 8

EXPNO : 8

PROCNO : 1

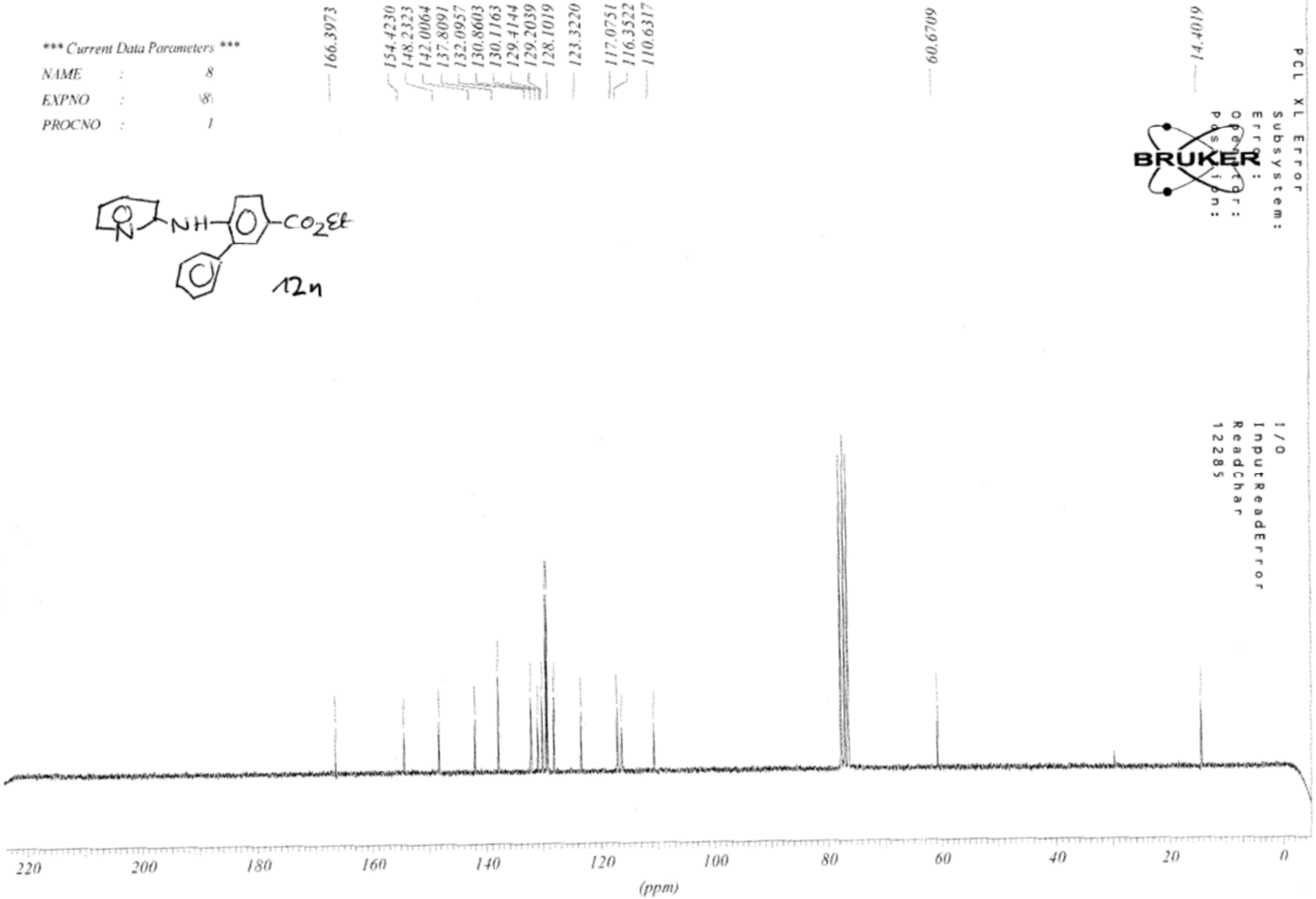

***N*-([1,1'-biphenyl]-2-yl)-3-methylpyridin-2-amine (12o):**

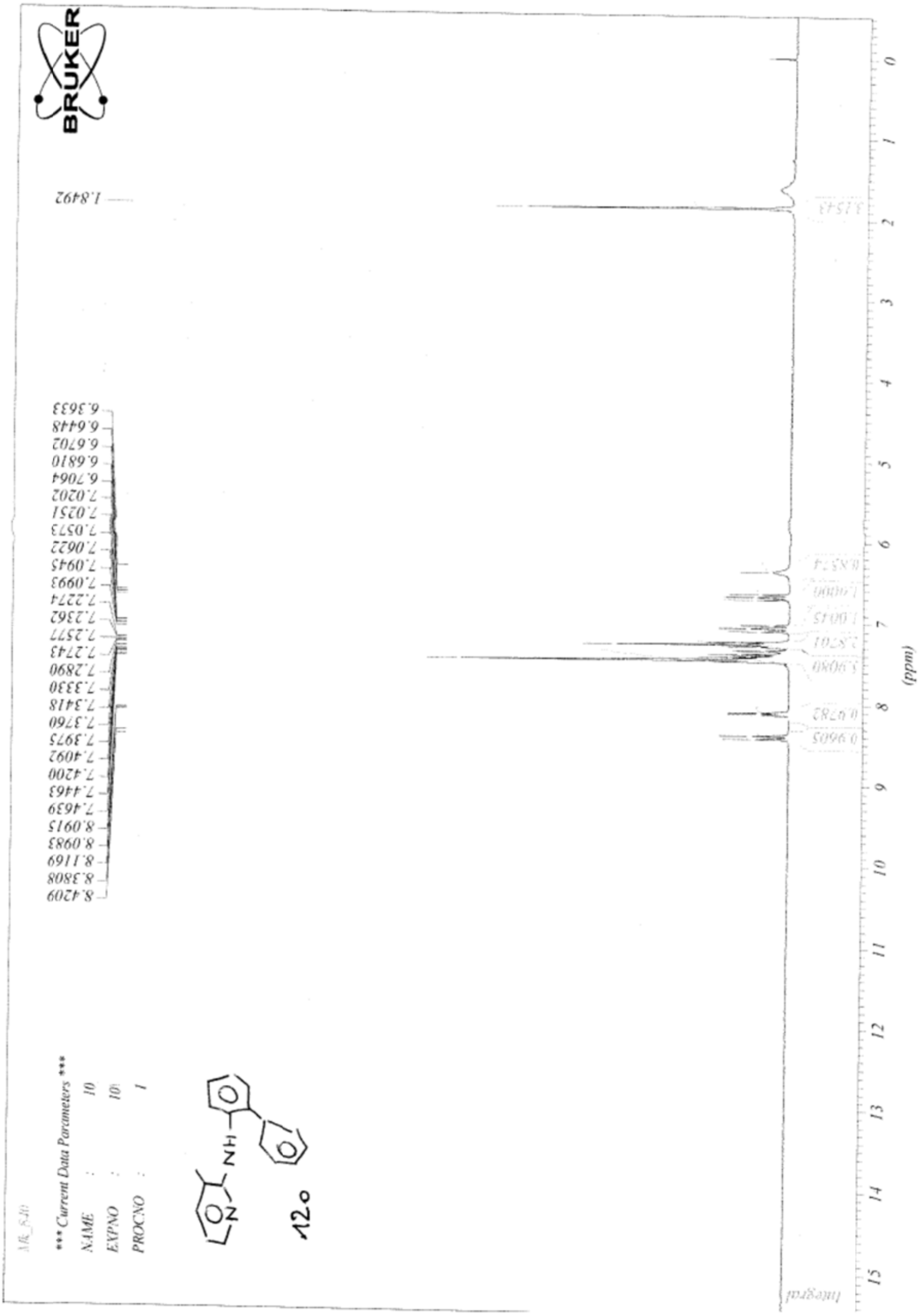

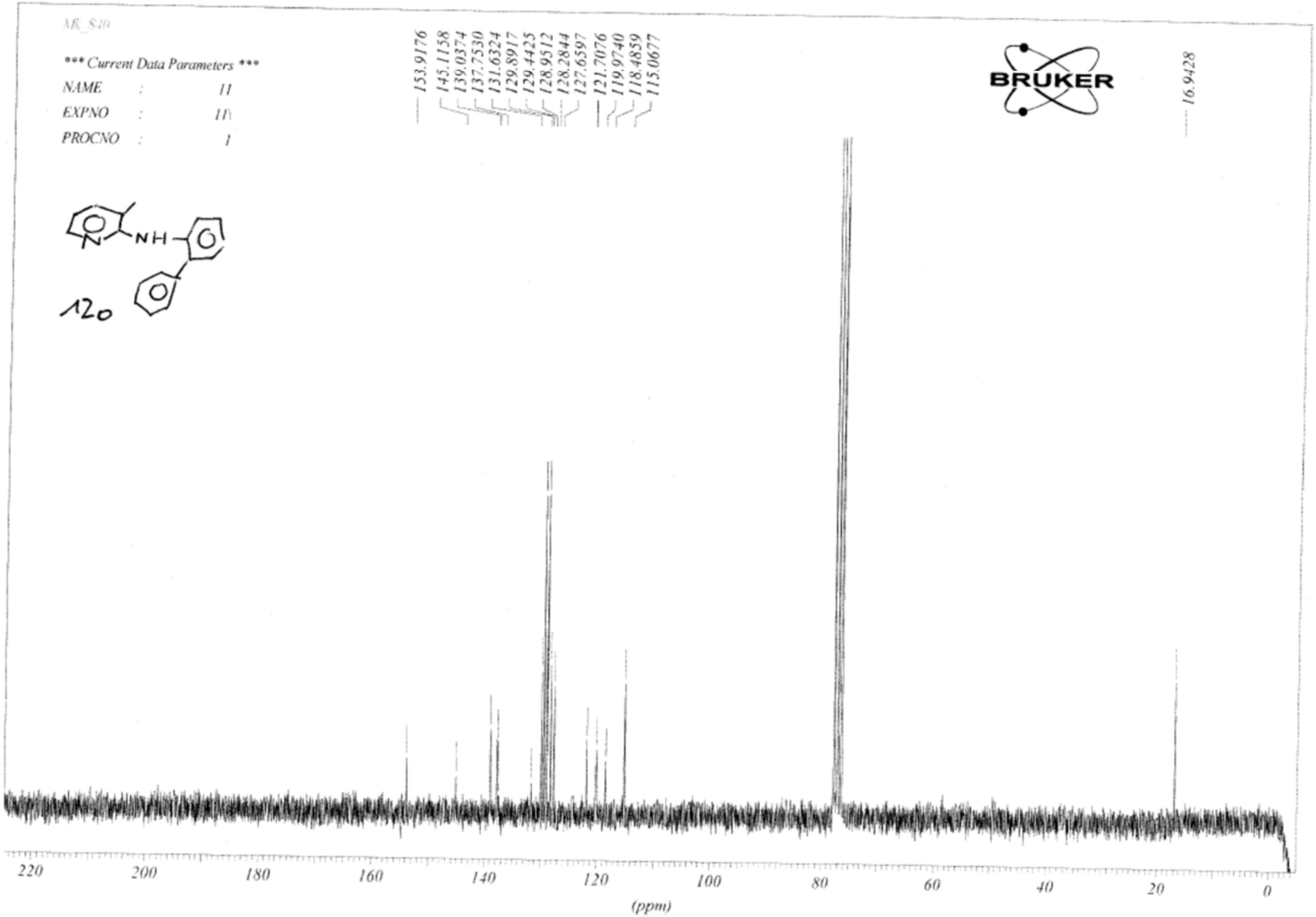
MK_840
*** Current Data Parameters ***
NAME : 11
EXPNO : 11
PROCNO : 1
NH
120
153.9176
145.1158
139.0374
137.7530
131.6324
129.8917
129.4425
128.9512
128.2844
127.6597
121.7076
119.9740
118.4859
115.0677
BRUKER
16.9428
220
200
180
160
140
120
100
80
60
40
20
0
(ppm)

N-([1,1':3',1''-terphenyl]-2'-yl)-3-methylpyridin-2-amine (13):

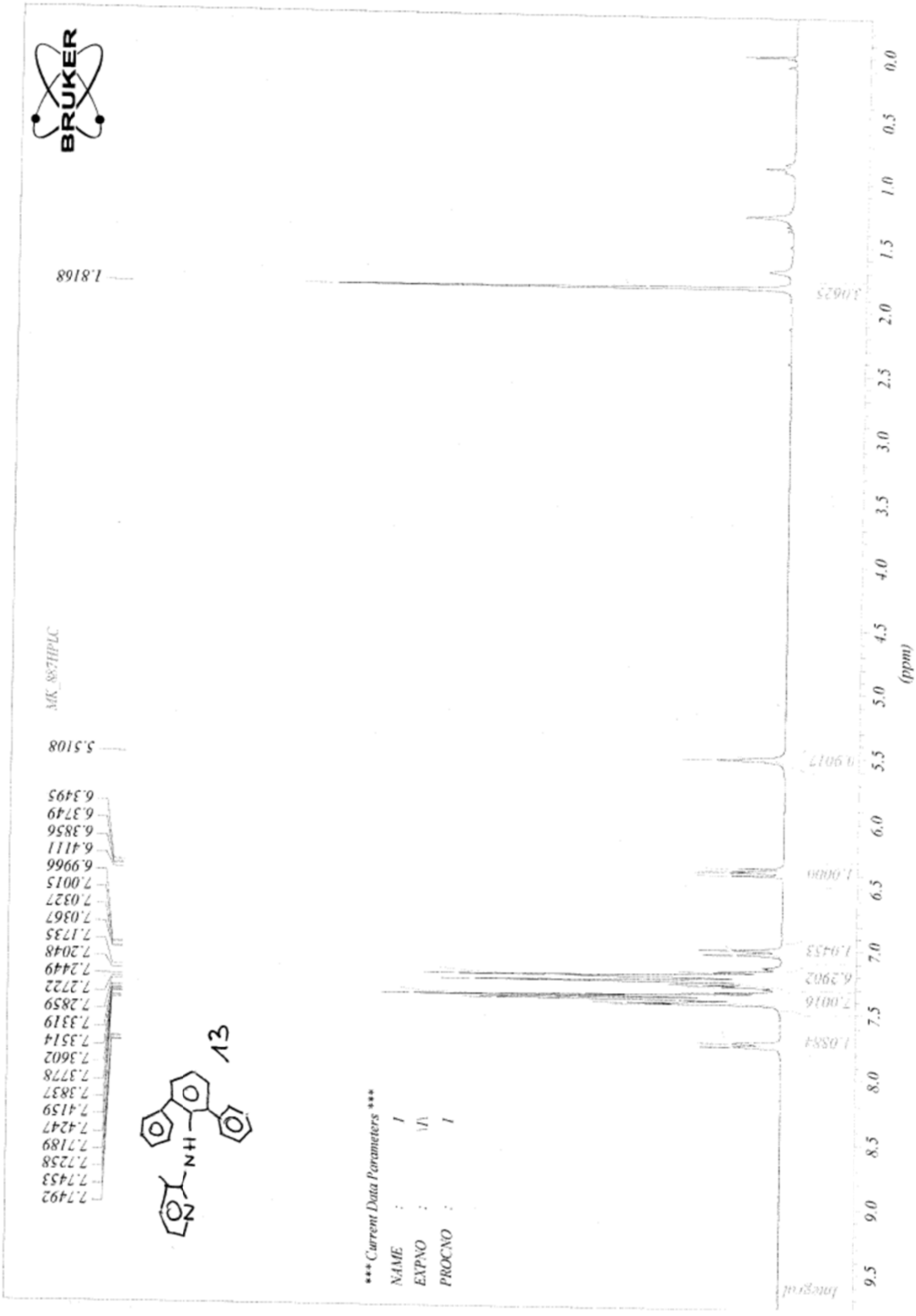

MK_882-HPLC

*** Current Data Parameters ***

NAME	:	21
EXPNO	:	21\
PROCNO	:	1

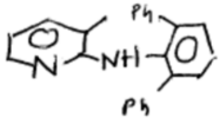

13

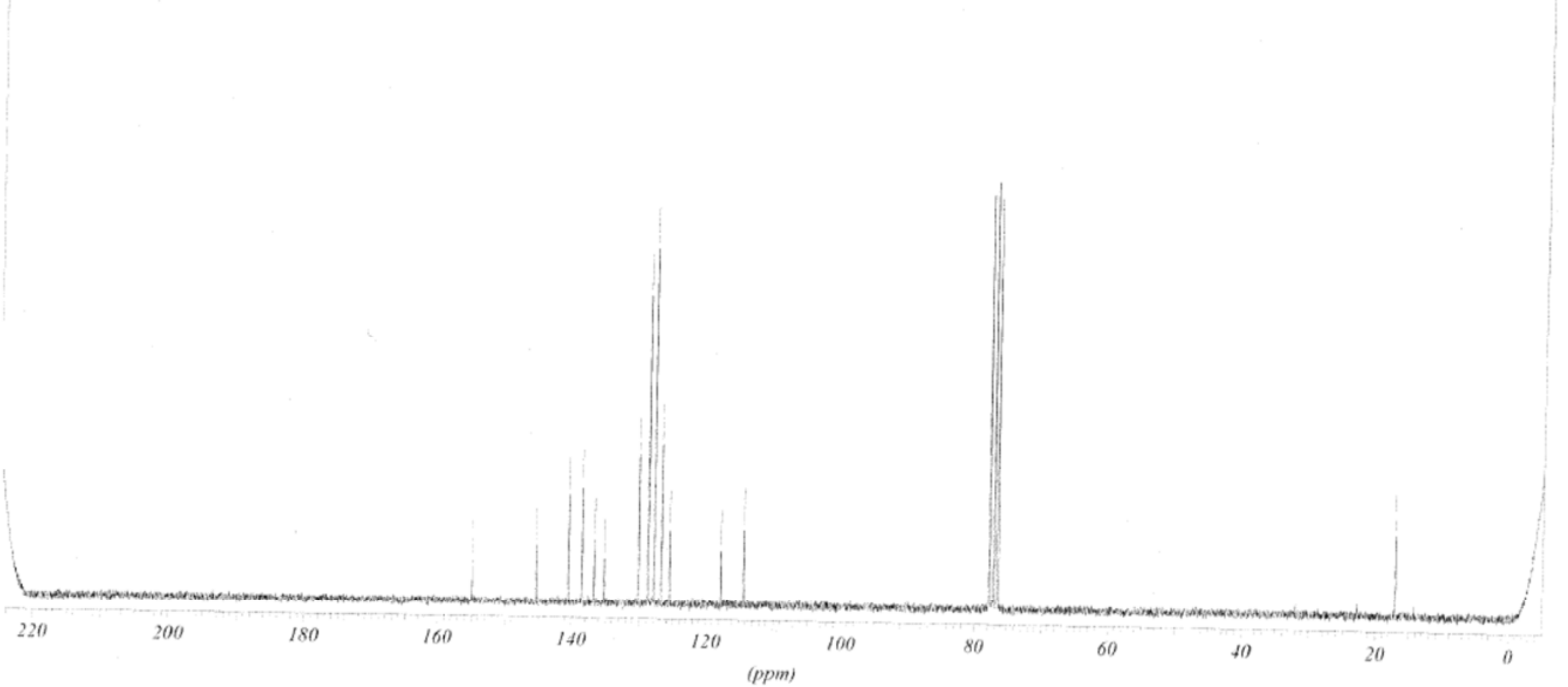

Supplement: Supplementary file 1 [file cctc0004-1345-SD1.pdf]
